# Physiological and anatomical responses to drought stress differ between two larch species and their hybrid

**DOI:** 10.1007/s00468-021-02129-4

**Published:** 2021-05-07

**Authors:** Nadia Sasani, Luc E. Pâques, Guillaume Boulanger, Adya P. Singh, Notburga Gierlinger, Sabine Rosner, Oliver Brendel

**Affiliations:** 1 Institute of Biophysics, University of Natural Resources and Life Sciences, Vienna, Austria; 2 INRAE, UMR BIOFORA, 45160 Ardon, France; 3 Université de Lorraine, AgroParisTech, INRAE, UMR Silva, 54000 Nancy, France; 4 Institute of Botany, University of Natural Resources and Life Sciences, Gregor Mendel Strasse 33, 1180 Vienna, Austria

**Keywords:** Constitutive wood anatomy, Drought stress, Hybrid larch, *Larix decidua*, *Larix kaempferi*, Reaction to drought, Water use efficiency

## Abstract

**Key Message:**

Hybrid saplings were more reactive to soil water deficit than Japanese and European larch. European larch had hydraulically safer wood and anisohydric behavior, Japanese and hybrid larch showed isohydric strategy.

**Abstract:**

Deciduous larch species could be an alternative to evergreen conifers in reforestation, but little is known about drought sensitivity of their saplings. The effect of an experimental drought on hydraulics and quantitative wood anatomy was tested on saplings of European larch (EL, *Larix decidua*), Japanese larch (JL, *Larix kaempferi*) and their hybrid (HL). Across species, biomass, transpiration rate and relative water content were higher in controls than in drought stressed trees, but transpiration efficiency was lower. JL had the highest transpiration efficiency under drought, and EL the lowest, coinciding with slower growth of EL. Wood of EL formed before drought was hydraulically safer as shown by higher wall/lumen ratio and lower pit cavity area. EL neither had a significant increase in transpiration efficiency nor a reduction in transpiration rate under drought, suggesting that the stomata remained open under soil water deficit. HL saplings were the most reactive to water shortage, indicated by intra-annual density fluctuations and a decrease in relative water content of the sapwood. Significant reduction in transpiration by HL suggested a higher stomatal sensitivity, while the same leaf surface area was maintained and radial growth was still similar to its best parent, the JL. The latter showed a significantly lower leaf surface area under drought than controls. EL, with its hydraulically safer wood, followed an anisohydric behavior, while JL and HL revealed an isohydric strategy. Altogether, our results suggest species dependent acclimations to drought stress, whereby HL followed the strategy of JL rather than that of EL.

**Supplementary Information:**

The online version contains supplementary material available at 10.1007/s00468-021-02129-4.

## Introduction

Climate change has drastic effects on the survival, reproduction, growth and productivity of trees. High temperatures together with water shortage are major stress factors, which cause physiological as well as structural changes (Breda et al. 2006; Allen et al. 2015; McDowell and Allen 2015; Mencuccini and Binks 2015; McDowell et al. 2016; Klein et al. 2019; Rozenberg et al. 2020). There is an expectation for forests to mitigate climate change through carbon storage and sequestration, protect against erosion, soil and snow slides; however, the demand for wood, particularly from conifers such as larch, has never been higher.

Species from the sub-family of the *Laricoideae*, European larch (*Larix decidua*) and Japanese larch (*Larix kaempferi*) are of high environmental and socio-economical importance in several parts of the northern hemisphere (Geburek 2002; Pâques et al. 2013; Caudullo et al. 2018). The native range of both species is mostly mountainous, but some populations of European larch grow at elevations lower than 400 m. Their cultivation range has been successfully extended north- and westwards mostly in oceanic and continental climates, including lowland areas across Europe (Geburek 2002; Pâques et al. 2013). With less than 700,000 ha across its native range, European larch is considered as a minor species among conifers but it plays a major ecological (protection against snow and soil slides) and economical (timber production) role at regional levels particularly in the Alps (Geburek 2002; Pâques et al. 2013). Larch wood is much appreciated for its high density, mechanical properties, natural durability (outdoor use) and the aesthetics (indoor use) of its heartwood (Pâques et al. 2013). The use of alpine European larch provenances for plantation in lowland regions resulted in local maladaptation at the beginning of the twentieth century (Jansen and Geburek 2016). Japanese larch, which proved less susceptible to European larch canker (*Lachnellula willkommii* Hartig), was thereafter used as an alternative but it failed in many locations due to its sensitivity to summer drought (Boudru 1986; Masson 2005). It is native to Japan and its natural distribution covers only about 390 km^2^, where it grows in elevations from 500 up to 2900 m. Japanese larch prefers cold temperate to subarctic climates with high rainfall in summer and snowy winters (Caudullo et al. 2018). Foresters and breeders discovered in the first years of the twentieth century that the hybrid between European and Japanese larches (*Larix *x* eurolepis*) was much more vigorous than either of the parental species. From then on larch hybrids have been bred for improvement of growth, stem form, wood quality or disease resistance (Schneck et al. 2002; Pâques et al. 2013). Besides a greater vigour, hybrid larch successfully combines favourable traits from both its parental species; e.g., resistance to larch canker and *Meria laricis* Vuill as well as fast juvenile growth from Japanese larch and stem straightness as well as finer branching from European larch (Pâques et al. 2013; Caudullo et al. 2018). While European larch prefers a more continental climate, plantations of Japanese larch are restricted to oceanic conditions (e.g*.,* Western France, UK, Ireland, Denmark, Belgium). In contrast, their inter-specific hybrid has proved suitable in a much wider spectrum of sites, ranging from maritime to continental and from sea level to low mountain ranges (Pâques et al. 2013; Greenwood et al. 2015). Hybrid larch can be thus successfully cultivated, wherever European or Japanese larches would have been cultivated. The use of larch or their hybrids outside their native ranges raises specific questions in terms of deployment and maladaptation. Facing the pressure of predicted climate change, an important goal to improve breeding of larch is to integrate drought responses from different species and their crosses.

The drought response of European larch has been extensively studied on mature trees (e.g., Anfodillo et al. 1998; Beikircher et al. 2010; Eilmann and Rigling 2012; Schuster and Oberhuber 2013; Swidrak et al. 2013; Dietrich et al. 2019), with the constraint that the level of drought stress can only be approximately determined. European larch has been considered as an anisohydric species (Swidrak et al. 2013), showing little stomatal response at the onset of drought and maintaining transpiration and carbon assimilation, while plant water potential is on the decrease (Streit et al. 2014). A high stomatal conductance during favourable conditions would optimise CO_2_ fixation and, therefore, carbon accumulation and biomass growth, but might unduly increase water losses due to transpiration, thus resulting in low intrinsic water use efficiency (WUE, ratio between net CO_2_ assimilation rate and stomatal conductance to water vapour). As a mature tree, European larch is known to have a lower WUE compared to other conifer species (Schuster and Oberhuber 2013). Similar observations were made by Anfodillo et al. (1998) on mature European larch trees in the Alps, where sap flow did not diminish despite an ongoing soil water deficit, by tolerating decreasing water potential. However, with increasing drought severity, sap flow strongly decreases and growth declines (Leo et al. 2014; Obojes et al. 2018). With a hydraulic safety margin lower than other native conifers, the decreasing plant water potential greatly increases the risk of xylem cavitation in European larch, as has been shown by Beikircher et al. (2010). Nevertheless, the study by Dietrich et al. (2019) on several tree species including European larch showed that after a severe drought period as in 2015, there was little indication of xylem embolism or carbohydrate depletion. Other studies indicated lower diameter growth of European larch during drought events than other native conifer species (Eilmann and Rigling 2012; Schuster and Oberhuber 2013; Lévesque et al. 2013, 2014a; Feichtinger et al. 2014; Streit et al. 2014).

Under the impact of drought, Japanese larch shows both isohydric and anisohydric strategies, with a stronger tendency towards isohydry (Bhusal et al. 2020). Japanese larch is rather drought sensitive (Boudru 1986; Masson 2005; Huang et al. 2017) and might be in danger when predicted climate change scenarios will take place; nevertheless, differences in growth response to drought do exist among provenances (Nagamitsu et al. 2018).

For hybrid larch, some reports exist, where the impact of drought on growth was investigated (Haasemann 1986; Cazaux et al. 1993; Marchal et al. 2019). Little information is, however, available on the strategy of hybrid larch to acclimate to drought. So far, we know that hybrid larch survival and growth are highly sensitive to soil water reserves (Cazaux et al. 1993). In a nursery experiment combining the two parental larch species and their hybrid, three types of soil and three water regimes, Haasemann (1986) observed the overall superiority for height growth at age two of the hybrid even in the drier situation. However, its growth was optimal in the treatment with an average water supply, whereas optimal conditions for Japanese and European larches were, respectively, the moistest and the driest water regimes. More recently, Marchal et al. (2019) compared the radial growth of mature trees across sites and along gradients of soil water availability: not only did the hybrid have superior growth—except at the lowest levels of water availability—but it also showed a higher phenotypic plasticity and overall better site stability than both parental species. The success of the hybrid over European and Japanese larches clearly depends on water availability, where soil water reserves below 75 mm become critical with more severe mortality and reduced growth (Cazaux et al. 1993). However, a better understanding of the behaviour of European, Japanese larch and their hybrid under soil water deficit is critical for deployment recommendations and breeding strategies.

For effective breeding strategies for drought resistance, detailed knowledge about a species’ ecological requirements during the whole rotation length will be necessary, especially as the hybrid might have inherited the drought sensitivity of Japanese larch (Boudru 1986; Masson 2005; Huang et al. 2017). The first years after plantation have been shown to be critical in tree development, in terms of resistance to drought in later stages and thus of the final plantation success (McDowell et al. 2008). Controlled greenhouse experiments on saplings allow simulation of drought stress and the direct comparison of the reaction of different species. As far as we know, drought stress experiments under controlled greenhouse conditions comparing different larch species or their crosses have not yet been performed.

The aim of our study was to characterise and to compare the drought response of saplings of two larch species, European larch and Japanese larch, and their hybrid under controlled drought conditions. The combination of these species was chosen, because European larch plays a major ecological role in, e.g., protection against snow and soil slides, and the hybrid of European and Japanese larch is of economical interest for timber production in plantations but we lack information on ecological requirements while in a juvenile state. Knowledge of their respective ecological requirements in the juvenile state would provide guidelines for their optimal deployment in forests. We address two main research questions: first, did the hybrid inherit drought sensitivity from the Japanese larch, and second, which anatomical traits are responsible for lower drought sensitivity? We hypothesise, that Japanese larch is more drought sensitive than European larch and that the hybrid shows higher drought plasticity during growth. Potential differences in drought sensitivity were examined with regard to whole plant transpiration response (plant water use, transpiration efficiency), biomass increase and wood formation. To infer hydraulic vulnerability, constitutive wood anatomy (anatomy before drought stress) including pit structural parameters was analysed. For the European larch, we hypothesise that its slower growth is associated with a hydraulically safer wood design, allowing a more anisohydric strategy, whereas a less safe design in the Japanese larch and the hybrid demands stronger stomatal control and thus a more isohydric strategy.

## Materials and methods

### Plant experimental setup

Three-year-old saplings of European larch (*Larix decidua* Mill., EL, seed source: Sudetan larch seed orchard Theil-FR), 4-year-old saplings of Japanese larch (*Larix kaempferi* (Lamb.) Carrière, JL, seed orchard Flensborg-DK) and 2-year-old saplings of hybrid larch (HL, *Larix* x *eurolepis*, parents of family REVE-VERT-FR) were used in our work. Due to different growth characteristics of the three species (especially the fast growth of the hybrid), saplings of similar age would have had very different sizes, whereas a selection purely on the size would have resulted in largely different ages. The tree height of European larch at the beginning of the experiment was 812.67 ± 101.67 mm, of hybrid larch 1236.50 ± 71.16 mm and of Japanese larch 1350.50 ± 88.77 mm. Prior to the experiment, plants were grown in two chambers of a glasshouse located at Champenoux, France (48°45′09.3″ N, 6°20′27.6″ E), under natural light conditions with daily maxima of irradiance ranging from 150 to 1000 µmol/m^2^/s PAR. Environmental conditions in the greenhouse were affected by weather conditions, but the temperature was maintained between 15 and 26 °C. Three plants from each group were grown at similar soil water content conditions with two different water regimes, respectively. The field capacity (FC) at planting was estimated at 30% ± 1.7% soil volumetric humidity (SVH). The automatic watering was adjusted to that; the control plants (C-trees) remained at 80% of the field capacity of the soil. This corresponds to 24% relative extractable soil water content (REW = (SVH-WP)/(FC-WP), considering a wilting point (WP) at 2% SVH). Forest trees are considered to be under drought constraint at relative water content below 40% relative extractable soil water content; therefore, a final target SVH for drought-stressed plants (S-trees) was below this value at 30% of the field capacity of the soil, corresponding to 10% SVH (Granier et al. 1999). The potted plants were put on a robotic weighing and watering system (Bogeat-Triboulot et al. 2019) after all trees had flushed on March 22, 2015 (day 80). The soil surface of each pot was covered with white marble gravel (diameter about 1 cm) to reduce soil water evaporation and four pots without plants, each for control and drought conditions, were used to estimate residual soil evaporation with the same frequency as the transpiration estimates for the plants. Cumulative transpiration over the whole experimental period (TRcum) was calculated by summing the water losses between two weighings of each plant. Plant transpiration rate (Trp, Online Resource 1) was calculated by dividing the water loss by the time between two weighings. The target drought level of 10% SVH was approached in a controlled stepwise way by not watering the pots until the target was reached. Bigger plants with a larger leaf surface use the available water reserve in the pots more rapidly, allowing more rapid approach of the final SVH target level. Thus, four levels of SVH (25%, 20%, 15%, 10%) were used and the advancement from one level to the next was constrained until all plants had reached this drought level; 25% SVH was reached on day 100, 20% on day 107, 15% on day 110 and 10% on day 160. The trees were under moderate drought conditions from day 107 to day 176, thus for 70 days. The number of irrigations per day was adjusted to the transpiration of the plants, so that overall each watering level was less than 100 ml water, starting with two irrigations per day at the beginning of the experiment, and five irrigations per day at the end. Plants were harvested on June 26, 2015 (day 176). For the determination of the relative water content (see below), a 5 cm long stem segment was cut just above the root collar. Stem samples with a length of 5 cm were collected adjacent to these segments and stored frozen at − 20 °C until the anatomical investigation. The sample set comprised three trees per treatment and species (*n* = 18, Online Resource 2).

### Biomass determination

To estimate initial biomass, allometric relationships were estimated from three individuals, representative of the size range, from each species. Stems and branches were measured for basal and apical diameters and length (overall *n* = 520). Roots were separated and washed. All parts were dried at 65 °C to constant mass and weighed. Dry mass (DM) was determined for each tree for each compartment (stem or branch) and volumes were summed up. Density was estimated as mass/volume, and an ANOVA analysis (separately for stem and branches) showed that there was no significant species effect for density and only a slight increment effect for the stem. It was, therefore, decided to estimate one allometry for stems, using a power equation (DM = 0.95145 × *V*^0.92404^, *R*^2^ = 0.94, *n* = 26) and one for branches (DM = 0.6569 × *V*^1.0008^, *R*^2^ = 0.92, *n* = 16). These allometries were applied to all measured stems and branches at the beginning of the experiment (*n* = 1105). Initial root biomass was estimated using the estimated stem mass (DM = 86.80 × stem – DM^0.642^; *R*^2^ = 0.66, *n* = 26). Stem, branch and root DM were used to calculate the initial dry mass of each tree.

During the final harvest, stems, branches and roots were separated. The roots were washed and all plant organs were dried as described above. For the harvested plants, the main stem, last year’s branches, new branches, needles from last year’s branches, needles from new branches and roots (washed) were separated and dried as indicated above. Final biomass was calculated as the sum of the dry masses of the different plant organs.

The biomass increment (BMinc) was calculated as the difference between the final biomass and the initial biomass (BMini). The relative biomass increment was calculated as BMinc/BMini and is given as a percentage.

### Needle surface area and plant transpiration per leaf surface area

To estimate final needle surface area (NSF), needles were harvested to establish an allometric relationship between needle dry mass (DM) and needle surface. The surface of all needles from three current year (2015) and three 1-year-old (2014) branches from each tree were measured using a flatbed scanner. ANOVA showed that there was neither a significant species effect nor a significant treatment effect on leaf mass per area. However, a significant needle age effect was detected. Therefore, we used two different allometric equations to convert needle dry mass into needle surface. Thus, power equations were established between needle mass and needle surface (for 1-year-old needles, NSF = 0.2358 × DM^0.8585^; for current year needles: NSF = 0.0145 × DM^1.0534^ with DM = needle dry mass). Total needle surface was estimated as the sum of the two needle generations (2014 and 2015).

### Transpiration traits

The assessment of the cumulative transpiration over the whole experimental period (TRcum) and the plant transpiration rate (Trp) is described in the section “Plant experimental setup”. The transpiration efficiency (TE) was calculated as the total biomass increase (BMinc) divided by the cumulative transpiration (TE = BMinc/TRcum).

Plant transpiration per hour and per leaf surface (TR) was calculated as the plant transpiration rate (Trp) divided by the total final needle surface (TR = Trp/NSF).

### Relative water content

The relative water content (RWC) of the trunk was estimated from a 5 cm section of the main stem just above the root collar. At the harvest, the bark was taken off and the fresh mass determined (FM), then the stem parts were infiltrated by keeping them under water in a closed glass container under vacuum, until air bubbles from stem parts had completely subsided. Then surface water was dried off and the water-saturated mass determined (SM). Subsequently, the samples were dried at 65 °C until constant weight to measure the dry mass (DM). RWC was calculated as RWC (%) = 100 × (FM-DM)/(SM-DM).

### Carbon isotope composition (*δ*^13^C)

At the final harvest, several needles from apical shoots were sampled, dried for 48 h at 70 °C and ground into a fine powder using a ball mill (Retsch GmbH, Haan, Germany). Subsamples of 1 mg ± 0.1 mg were weighed into tin capsules and the carbon isotopic composition was measured with an isotope ratio mass spectrometer (Thermo-Finnigan, Delta S, Bremen, Germany). The carbon isotope composition (*δ*^13^C) was calculated according to the international standard (Vienna Pee Dee Belemnite, VPDB) using the following equation: *δ*^13^C = (*R*_s_ – *R*_std_)/*R*_std_ × 1000, where *R*_s_ and *R*_std_ are the isotopic ratios ^13^C/^12^C of the sample and the standard, respectively. The precision of spectrometric analysis (standard deviation of *δ*^13^C) was assessed with a calibrated, internal laboratory reference material with a matrix close to the measured samples (*n* = 16, SD = 0.05‰).

### Wood formation of control and drought stressed trees (tracheidograms)

To evaluate the impact of drought on anatomical traits, radial lumen diameters and the tangential double cell wall thickness were measured in one complete radial cell row of the latest wood increment, for each tree. Dissected pieces of normal wood (avoiding compression wood) harvested at 5 cm from the ground were mounted in the sample holder of a cryo-microtome (CM 3050 S, Leica Biosystems Nussloch GmbH, Germany) keeping the orientation perpendicular to the main fiber axis. Disposable microtome blades (N35HR Blade 35, Feather, Japan) were used to cut 10–20-µm-thick transverse sections. Sections were stained with safranine/astra-blue. Lignified cell walls appear red after safranine staining, and non-lignified cell walls acquire blue colour after astra-blue staining. For anatomical analysis, sections were dehydrated in ethanol and mounted on slides in Canada balsam. Images were acquired with a Leica DM4000 M microscope equipped with a Leica DFC 320 R2 digital camera. Leica IM 500 image manager analyzing software was used for digital stitching (Leica, Weltzlar, Germany) and Image J software (https://imagej.nih.gov) for quantitative anatomical measurements. Mean values of radial lumen diameters and the tangential double cell wall thickness were thereafter calculated for 5% radial distance steps, with the whole wood increment as 100% reference. Means of anatomical traits measured in the region of 90–95% of the increment (radial lumen diameter, *b*_r_ 90–95% and tangential cell wall thickness, *t*_t_ 90–95%) were used for comparisons within and among species. The cell walls were still thickening in the region between 95 and 100% of the increment; we thus avoided analysing this part statistically.

### Constitutive wood anatomy: tracheid dimensions and quantitative pit anatomy

We refer “constitutive wood anatomy” to quantitative anatomical traits before plants were impacted by drought. This approach is based on Rosner et al. (2016) who found that the wood formed before drought stress impacts the sensitivity to drought in conifers. To elucidate whether the variation in anatomical functional traits, such as tracheid and pit dimensions, affected species’ performance of the trees under drought stress, we assessed such traits in the first formed early wood tracheids of the latest annual ring. ANOVA indicated no influence of initial biomass and treatment (drought stress) on the traits investigated, indicating that before stress started, no anatomical differences were present which were related to the size of the saplings or treatment. Therefore, statistical analysis on species differences of tracheid dimensions, conduit wall reinforcement and pit traits were done by pooling all trees from a given species.

Tracheid dimensions and conduit wall reinforcement were assessed on wood sections stained with safranine/astrablue (details in an earlier section). Double cell wall thickness (*t*) and lumen diameter (*b*) of early wood cells were measured in radial (*t*_r_, *b*_r_) and tangential (*t*_t_, *b*_t_) directions in the first 10 cell rows of the 2015 growth ring using Image J software. For each of the four traits, a tree mean value was calculated from 20 single measurements that were performed on four different positions around the whole circumference. Conduit wall reinforcement was calculated in the radial ((*t*_r_/*b*_r_)^2^) and tangential ((*t*_t_/*b*_t_)^2^) direction (Rosner et al. 2018).

**Fig. 1:**
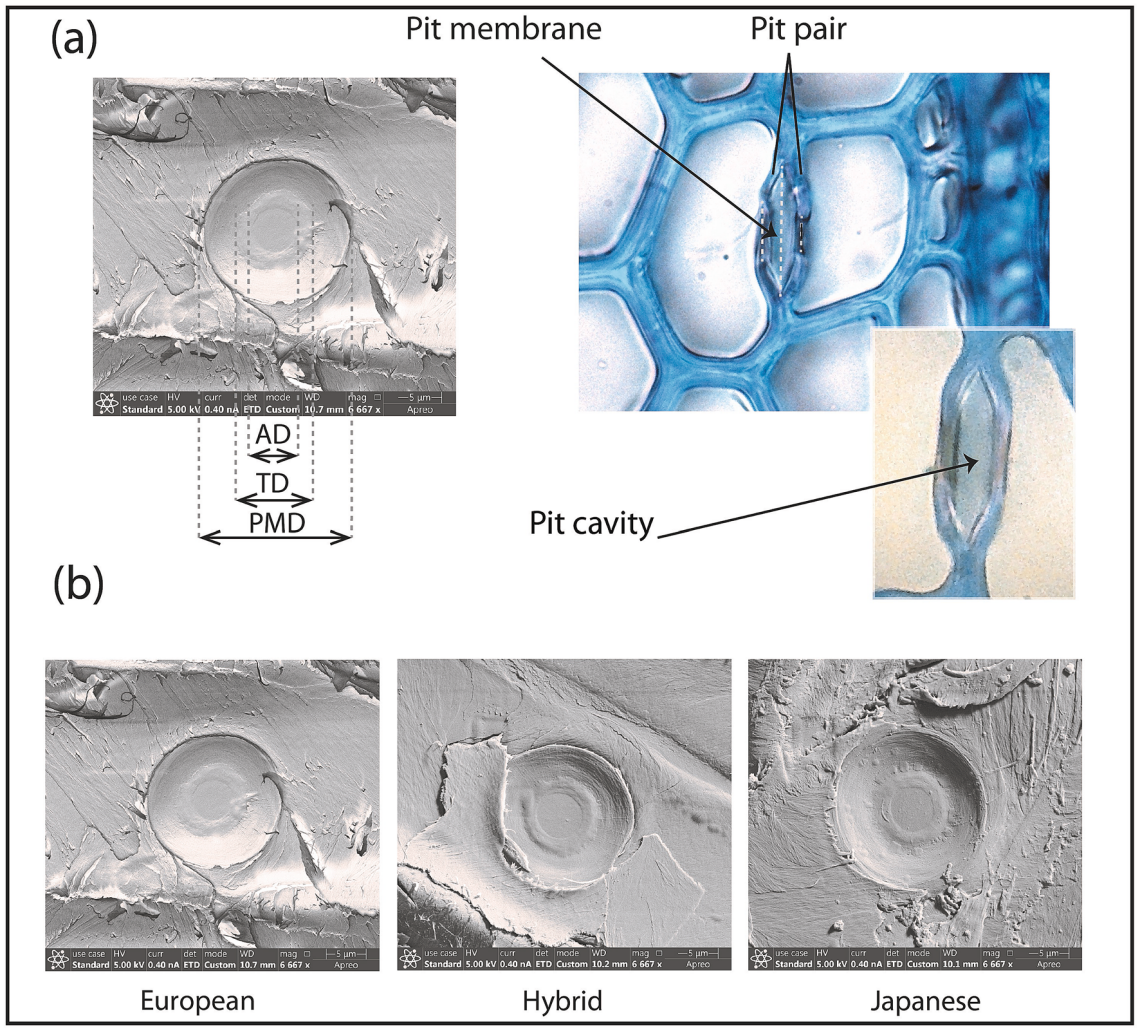
Documentation of measurements on bordered pits. **a** left side, SEM image of a radial wood section showing a bordered pit with pit aperture diameter (AD), torus diameter (TD) and pit membrane diameter (PMD). **a** right side, light microscopy photo of a transverse wood section stained with toluidine blue; the insert shows the pit cavity at higher magnification. **b** SEM images of bordered pits of European, hybrid and Japanese larch

Light microscopy images were used for the determination of the pit cavity area (Fig. 1a, right). SEM images (Fig. 1a, left; Fig. 1b) were used for all other measurements, such as the pit membrane diameter (PMD) torus diameter (TD) and the pit aperture diameter (AD).

For light microscopy, sections (5–10 µm thick) were cut from frozen samples using a cryo-microtome. Preliminary work undertaken to select the most effective stain indicated that toluidine blue, which stains wood cell wall, including the pit border, greenish blue and the pit torus pink–magenta, to be the most suitable stain. The sections were stained for 2 min, then washed with water and mounted on glass slides in a drop of water. Nail polish was used to seal the cover glass. The sections were examined with a light microscope (Nikon, Japan) and the images were captured with a digital camera. For each sample, 20 intact pits were selected in early wood of the 2015 wood increment. Pit cavity was defined and measured using image J (Fig. 1a, right).

For SEM, air-dried wood blocks were split longitudinally, exposing the radial face. The samples were mounted on stubs and coated with gold particles in a sputter coater (LEICA EM SCD005). Images were acquired using the Apreo SEM (ThermoFisher Scientific, Massachusetts, USA) at 5 kV. Measurements of membrane diameter (PMD), torus diameter (TD) and pit aperture diameter (AD) were done using the ImageJ software. Twenty pits were used for each tree sample. Torus overlap (TO, torus–aperture overlap, torus to aperture ratio) was determined as (TD-AD)/TD (Fig. 1a, left).

### Statistical analyses and sample numbers

Traits investigated and analysed as well as their abbreviations are listed in Table 1. Differences between species and the drought treatment effects of biomass- and transpiration-related traits were analysed using an ANCOVA model (R Core Team 2020) in particular for taking into account the different sizes of trees at the beginning of the experiment:

$$Y = {\text{ BMini }} + {\text{ species }} + {\text{ treatment }} + {\text{ species }} \times {\text{ treatment}},$$

**Table 1:** List of investigated growth parameters, physiological- and anatomical traits and their abbreviations

Abbreviation	Trait	Unit
AD	Aperture diameter of bordered pits	µm
BMinc	Biomass increase	g
BMinc%	Relative biomass increase	%
BMini	Initial biomass	g
*b*_r_	Radial lumen diameter of tracheids	µm
*b*_r_ 90–95%	*b*_r_ in 90–95% of the radial increment 2015	µm
*b*_*t*_	Tangential lumen diameter of tracheids	µm
DS	Diameter of the stem	mm
PC	Pit cavity	µm^2^
PMD	Pit membrane diameter	µm
RWC	Relative water content	%
NSF	Needle surface area	cm^2^
TD	Torus diameter	µm
TE	Transpiration efficiency	g/kg
TO	Torus overlap	%
*t*_r_	Radial double cell wall thickness	µm
(*t*_r_/*b*_r_)^2^	Conduit wall reinforcement radial direction	
TR	Transpiration rate per needle surface	g/h/m^2^
*t*_t_	Tangential double cell wall thickness	µm
*t*_t_ 90–95%	*t*_t_ in 90–95% of the radial increment 2015	µm
(*t*_*t*_/*b*_*t*_)^2^	Conduit wall reinforcement tangential direction	
*δ*^13^C	Carbon isotope composition current year shoots	‰

BMini is the covariate for initial biomass differences among plants, species is the effect for European, hybrid or Japanese larch, treatment is the control vs. drought effect and species × treatment is their interaction (only included in the model when significant). BMini was only significant (*P* < 0.05) for few traits (TR, TRcum, TE, NSF, BMinc%). The normality of the residuals of the ANCOVA analyses was tested using the Shapiro–Wilks test. We detected a significant deviation from normality for TR and Trcum. A Box–Cox transformation was applied. As the ANOVA on the transformed variables resulted in similar significance levels for the different factors, it was decided to only present the untransformed results. Means and group differences for species, treatment and species × treatment were estimated using the HSD test function (Tables 2, 3 and 4, HSD.test, agricolae package, R Core Team 2020) to reflect the measured data. However, when an influence of the initial biomass was observed, within factor significant pairwise differences were estimated using also marginal means (emmeans, emmeans package, R Core Team 2020).

**Table 2:** Means and standard errors of physiological traits and biomass parameters for the three species, as well as for control vs. drought plants; letters are representing significant differences in the measured means and marginal means

Trait	Unit	BMini	Species	European larch	Hybrid larch	Japanese larch	Treatment			Int
		*P*	*P*				*P*	Control	Drought stressed	*P*
TR	g/h/m^2^	*	–	0.0116 ± 0.0008a	0.0107 ± 0.0024a	0.0076 ± 0.0009b	***	0.0128 ± 0.0010a	0.0071 ± 0.0009b	**
				0.0086 ± 0.0011A	0.0105 ± 0.0005A	0.0107 ± 0.0012A		0.0130 ± 0.0004A	0.0068 ± 0.0004B	
TE	g/kg	–	**	4.01 ± 0.33c	4.90 ± 0.46b	6.15 ± 0.81a	***	3.92 ± 0.21b	6.13 ± 0.50a	*
*δ*^13^C	‰	–	**	– 27.89 ± 0.40b	– 27.45 ± 0.44ab	– 27.05 ± 0.44a	***	– 28.37 ± 0.18b	– 26.55 ± 0.15a	–
RWC	%	–	–	95.9 ± 1.7a	90.8 ± 3.1a	91.0 ± 1.3a	**	96.0 ± 1.0a	89.1 ± 1.9b	–
NSF	cm^2^	***	–	1594 ± 295c	2605 ± 311b	4617 ± 544a	**	3536 ± 601a	2342 ± 380b	–
				3312 ± 396A	2692 ± 189A	2813 ± 411A		3370 ± 158A	2508 ± 158B	
BMini	g	–	***	61.2 ± 4.1c	108.5 ± 8.4b	163.3 ± 8.5a	–	115.9 ± 17.4a	106.2 ± 13.8a	–
BMinc	g	–	***	106.9 ± 14.5c	190.0 ± 28.8b	309.5 ± 34.0a	***	255.4 ± 37.2a	148.8 ± 23.5b	*
BMinc%	%	***	**	172.4 ± 15.8a	186.0 ± 40.0a	187.7 ± 13.8a	***	225.4 ± 18.3a	138.7 ± 7.6b	–
				76.8 ± 25.3C	181.2 ± 12.1B	288.0 ± 26.3A		234.6 ± 10.1A	129.5 ± 10.1B	
DS	mm	–	–	17.7 ± 1.1c	21.7 ± 1.2b	24.8 ± 1.7a	***	24.1 ± 1.3a	18.7 ± 1.0b	–
*b*_r_ 90–95%	µm	–	***	22.17 ± 2.34a	12.59 ± 2.24b	13.72 ± 2.06b	***	20.59 ± 1.85a	11.73 ± 1.56b	–
*t*_t_ 90–95%	µm	–	*	4.03 ± 0.43b	6.33 ± 0.68a	6.09 ± 0.76ab		4.85 ± 0.52a	6.12 ± 0.64a	–

Marginal means can be found below the measured means in case there was a significant effect of initial biomass. Significant differences in the mean values at the *P* < 0.05 level for either species or treatment are indicated by different letters, for marginal means by capital letters. Abbreviations are explained in Table 1

Results of ANCOVA analysis including effects of initial biomass (BMini), treatment and interaction (Int) factors are indicated with “-” for *P* ≥ 0.5, “*” for *P* < 0.05, ** for *P* ≤ 0.01 and “***” for *P* ≤ 0.001, n = 6 trees/species and 9 trees/treatment

We investigated three trees per species and treatment, respectively. In total, 18 trees were investigated (Online Resource 2). For analyses of constitutive wood- and pit anatomy, six trees per species were pooled. Tree mean values for constitutive wood- and pit anatomy were calculated from 20 single measurements per tree, respectively. Final statistical analyses were then performed with tree mean values.

## Results

### Effects of initial biomass, species and drought and their interaction on growth and physiology

**Fig. 2:**
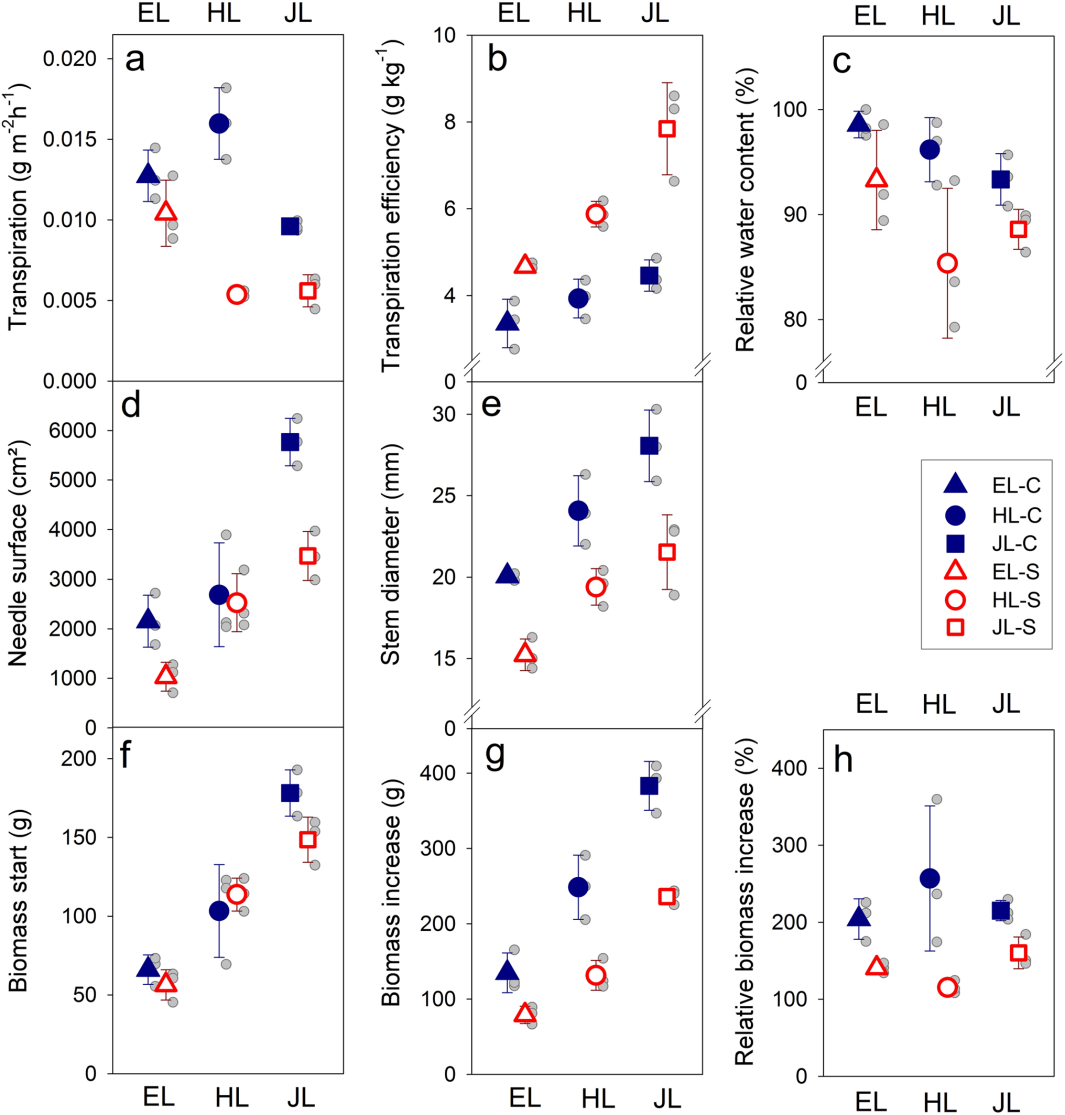
Transpiration rate per needle surface (**a**), transpiration efficiency (**b**), relative water content (**c**), needle surface area (**d**), stem diameter at the beginning of the experiment (**e**), biomass at the beginning of the experiment (**f**), biomass increase (**g**) and relative biomass increase (**h**) of three different larch species. Whiskers indicate the standard deviation and grey dots data for each single tree. Closed symbols indicate “control” trees, open symbols indicate drought stressed trees. *EL* European larch; *HL *hybrid larch, *JL* Japanese larch

Significant species effects were found for biomass at the beginning of the experiment (Fig. 2f). Therefore, to take this into account in the following analyses, initial biomass was tested as a covariate. Transpiration rate, needle surface area and relative biomass increase showed a significant effect of the initial biomass (Table 2).

Significant species effects were found for absolute- and relative biomass increase, transpiration efficiency and *δ*^13^C, with JL having the highest values, followed by HL and EL. No significant overall species effect was found for transpiration rate, RWC, needle surface area and stem diameter (Table 2).

A significant drought effect was found for all traits except for initial biomass, indicating an even distribution of plants across treatments before the stress was applied (Table 2).

Species*treatment interaction effects were significant for all transpiration traits and the absolute biomass increase (Table 2).

Information on the impact of drought within and among species on physiological and growth traits can be found in Table 3. Transpiration rate (Fig. 2a) and transpiration efficiency (Fig. 2b) had a significant drought effect in HL and JL (marginal means, Table 3). Drought stressed HL had a similar transpiration rate as JL, but it showed a much stronger decline in transpiration rate than JL (Table 3, Fig. 2a) and a lower increase of transpiration efficiency (Table 3, Fig. 2b). Within species, *δ*^13^C was significantly higher (less negative) in drought stressed than in control trees (Table 3). RWC was also lower in stressed trees than in control trees (Fig. 2c), but a significant decrease was solely found for HL (Table 3). Needle surface area (Fig. 2d) was significantly lower in stressed JL; EL showed a similar trend, whereas HL had similar measured values in both groups (Table 3). Absolute biomass increase (Fig. 2g) was significantly lower in HL and JL. Even though relative biomass increase (Fig. 2h) was affected by the initial biomass (Table 2), we found a significant decrease for the marginal means of drought stressed HL and JL (Table 3). Stem diameter was significantly affected by drought in all three species (Fig. 2e, Table 3).

**Table 3:** Mean values and standard errors of physiological traits and biomass parameters for interaction of species and treatment (C Control, S Drought stress)

Trait	Units	BMini *P*	European C	European S	Hybrid C	Hybrid S	Japanese C	Japanese S
TR	g/h/m^2^	*	0.0127 ± 0.0009b	0.0104 ± 0.0012b	0.0160 ± 0.0013a	0.0054 ± 0.0001c	0.0096 ± 0.0002b	0.0056 ± 0.0006c
			0.0101 ± 0.0011BC	0.0072 ± 0.0013BC	0.0155 ± 0.0007A	0.0055 ± 0.0007B	0.0136 ± 0.0015AC	0.0078 ± 0.0010B
TE	g/kg	–	3.36 ± 0.32c	4.67 ± 0.04bc	3.93 ± 0.26c	5.88 ± 0.17b	4.46 ± 0.21bc	7.84 ± 0.61a
*δ*^13^C	‰	–	– 28.74 ± 0.24c	– 27.03 ± 0.17ab	– 28.44 ± 0.14c	– 26.47 ± 0.06a	– 27.94 ± 0.39bc	– 26.16 ± 0.18a
RWC	%	–	98.6 ± 0.7a	93.3 ± 2.7ab	96.2 ± 1.8a	85.4 ± 4.1b	93.3 ± 1.4ab	88.6 ± 1.1ab
NSF	cm^2^	***	2156 ± 303 cd	1032 ± 169d	2686 ± 603bc	2525 ± 337bc	5767 ± 276a	3468 ± 284b
			3383 ± 438AB	2525 ± 502AB	2897 ± 263AB	2451 ± 257AB	3934 ± 589A	2444 ± 392B
BMini	g	–	66.1 ± 5.4c	56.4 ± 5.6c	103.3 ± 17.0bc	113.8 ± 6.0b	178.2 ± 8.5a	148.6 ± 8.3ab
BMinc	g	–	134.8 ± 1.5c	78.9 ± 6.6c	248.5 ± 24.6b	131.5 ± 11.4c	382.9 ± 18.8a	236.1 ± 5.7b
BMinc%	%	***	204.2 ± 15.1ab	140.6 ± 3.7bc	256.8 ± 54.4a	115.3 ± 4.7c	215.1 ± 7.6ab	160.2 ± 12.0bc
			122.2 ± 29.8ABC	40.9 ± 34.1BC	242.7 ± 17.9DE	120.2 ± 17.5B	337.5 ± 40.0AD	228.6 ± 26.6CE
DS	mm	–	20.1 ± 0.1bc	15.2 ± 0.6d	24.1 ± 1.2ab	19.4 ± 0.6 cd	28.1 ± 1.3a	21.5 ± 1.3bc
*b*_r_ 90–95%	µm	–	27.1 ± 0.4a	17.2 ± 1.6b	17.5 ± 0.4b	7.7 ± 1.1c	17.1 ± 2.9b	10.3 ± 1.1c
*t*_t_ 90–95%	µm	–	3.8 ± 0.7a	4.3 ± 0.5a	5.2 ± 0.1a	7.4 ± 1.0a	5.6 ± 1.3a	6.6 ± 0.9a

Marginal means are provided below the measured means when there was a significant effect of initial biomass. Significant differences in the mean values at the *P* < 0.05 level for either species or treatment are indicated by different letters, for marginal means by capital letters

Abbreviations are explained in Table 1

For comparison, significant biomass effects from the ANCOVA (BMini *P*), shown already in Table 2, are indicated with “-” for *P* ≥ 0.5, “*” for *P* < 0.05, ** for *P* ≤ 0.01 and “***” for *P* ≤ 0.001, *n* = 3 trees/species/treatment

### Characteristics of wood produced under the impact of drought

**Fig. 3:**
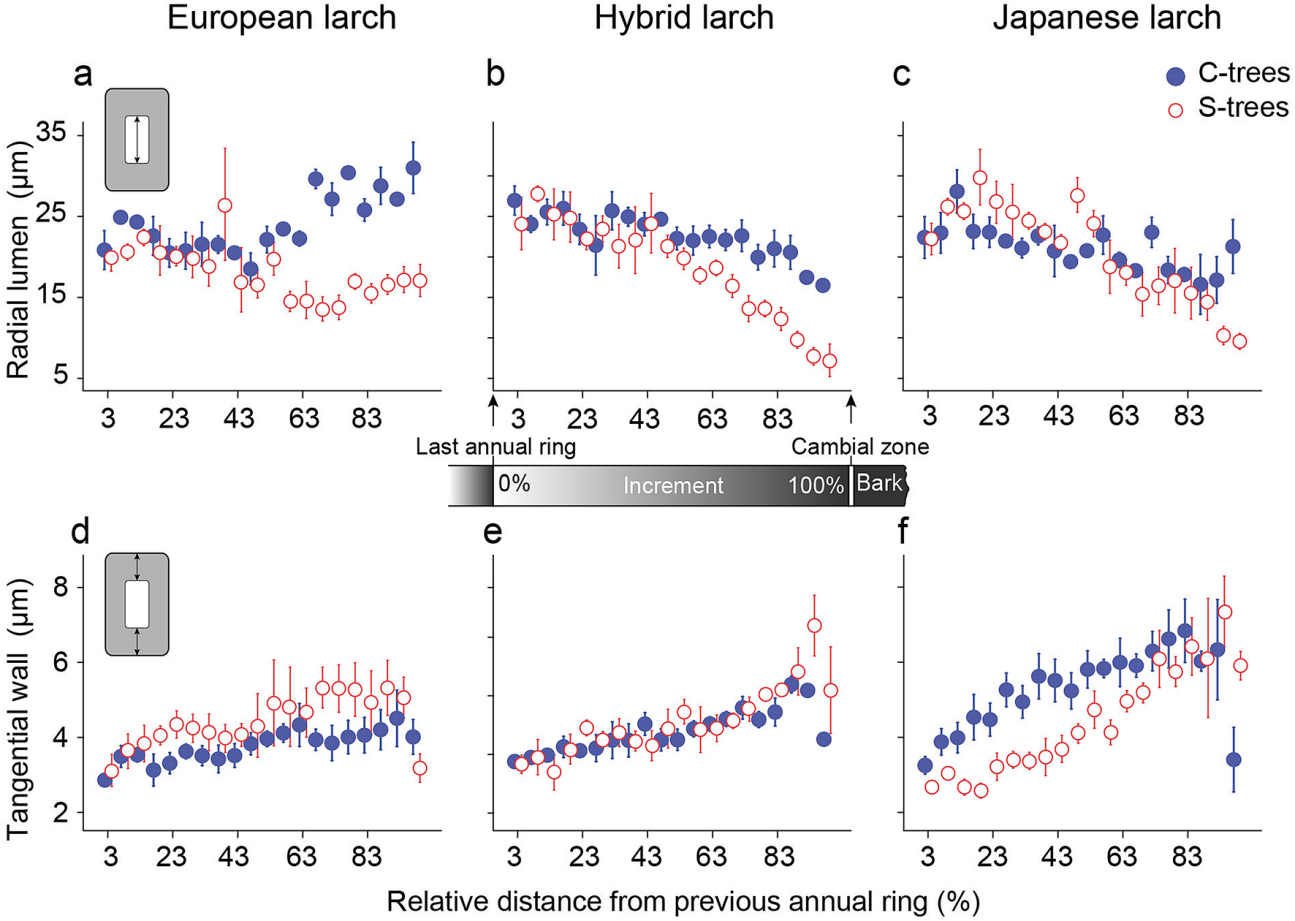
Tangential wall thickness and radial lumen diameter in 5% steps of the 2015 increment of European, hybrid and Japanese larch trees with two treatments (control and drought stress). Each stepwise mean value of radial lumen diameter of European larch (**a**), hybrid larch (**b**), and Japanese larch (**c**), and tangential wall thickness of European larch (**d**), hybrid larch (**e**), and Japanese larch (**f**) is shown with standard deviation for the two treatments “control” (C-trees, closed symbols) and drought stressed trees (S-trees, open symbols). Whiskers indicate the standard deviation

**Fig. 4:**
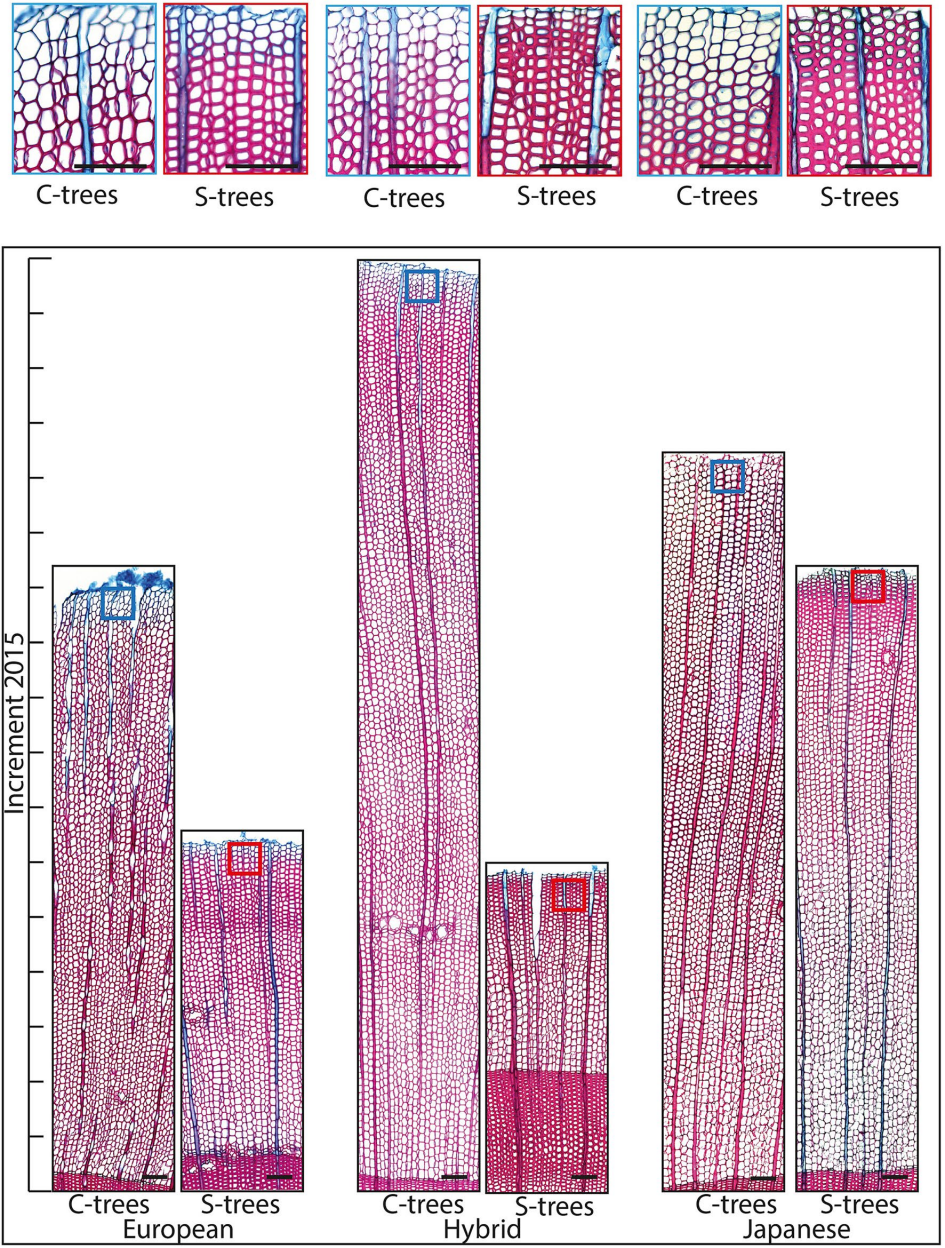
Selected transverse sections of the wood increment 2015 of control trees (C-trees) and drought stressed trees (S-trees) of European larch (EL), hybrid larch (HL) and Japanese larch (JL). Boxed regions show the rear part of the increment in higher magnification. Sections were stained with safranin and astrablue. The scale bar is 100 µm

**Fig. 5:**
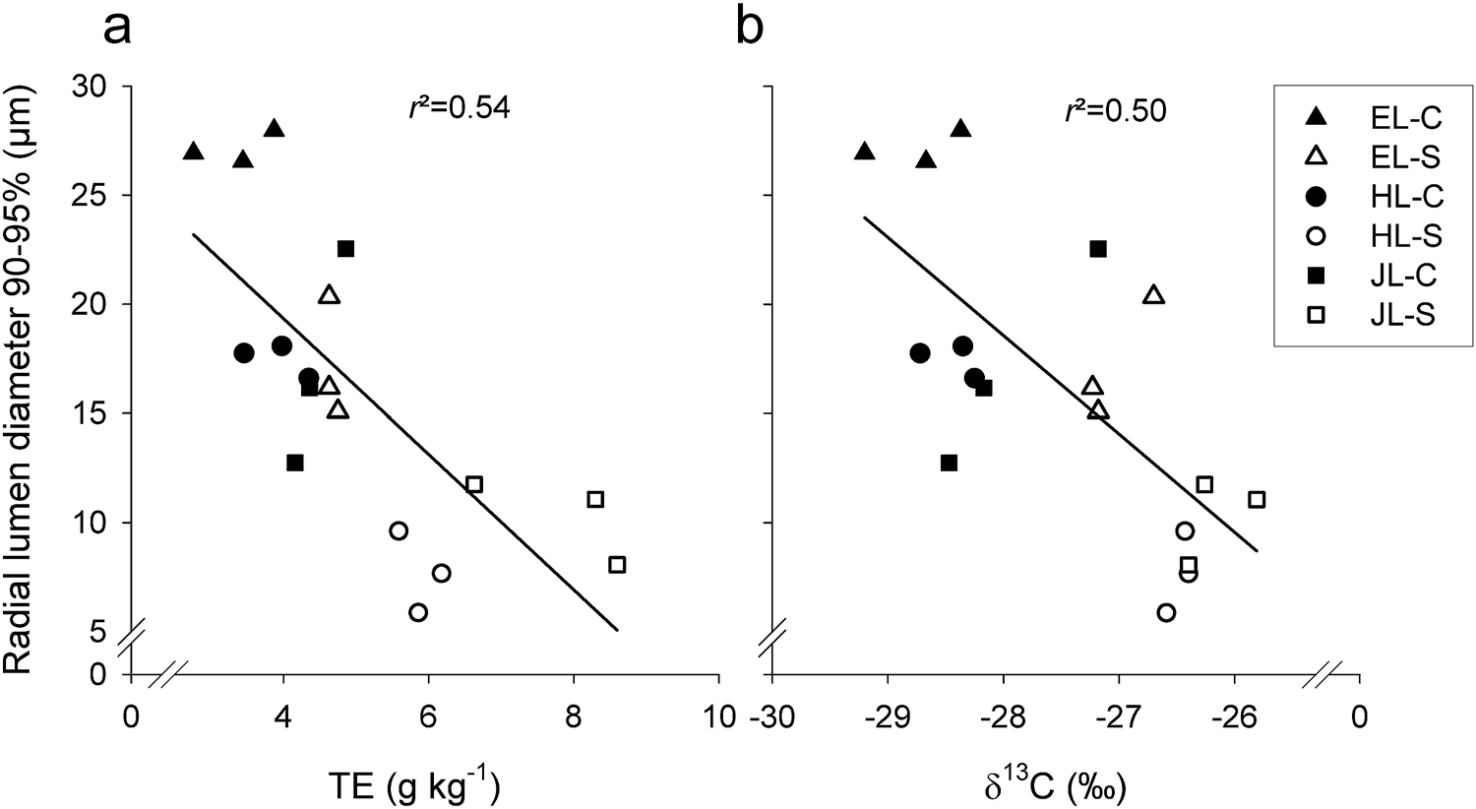
Relationships between radial lumen diameters in 90–95% of the latest formed annual ring and physiological traits such as transpiration efficiency (**a**) and *δ*^13^C (**b**). Triangles indicate European larch (EL-C, EL-S), circles hybrid larch (HL-C, HL-S) and squares Japanese larch (JL-C, JL-S) of control (closed symbols, EL-C, HL-C, JL-C) and drought stressed (open symbols, EL-S, HL-S, JL-S) trees, respectively

Differences in wood anatomy were analysed in 5% steps along the latest radial increment. Stressed trees showed a decrease in radial lumen starting at about 50% of the increment for all species (Fig. 3). When lumen diameters were plotted against the absolute distance from pith to bark (tracheidograms), the extreme reaction of HL to drought stress became obvious (Online Resource 3): in addition to a decrease in radial increment, lumen diameters decreased (see also examples in Fig. 4). In the region of the last 90–95% of the increment, radial lumen diameters significantly decreased due to drought in all species, but drought stressed EL had significantly larger lumens than stressed HL and JL (Table 3). Cell wall thickness in 90–95% of the increment also showed a tendency (treatment factor in ANOVA *P* = 0.08, Table 2) for higher values under drought, especially in HL (Table 3, Fig. 3). Smaller lumen diameters together with an increase in cell wall thickness in stressed trees resulted in a denser wood produced towards the end of the experiment (Fig. 3). We found negative correlations between radial lumen diameters in 90–95% of the increment and transpiration efficiency (Fig. 5a) as well as with carbon isotope compositions of the current year shoot (*δ*^13^C) (Fig. 5b).

### Constitutive wood anatomy

**Fig. 6:**
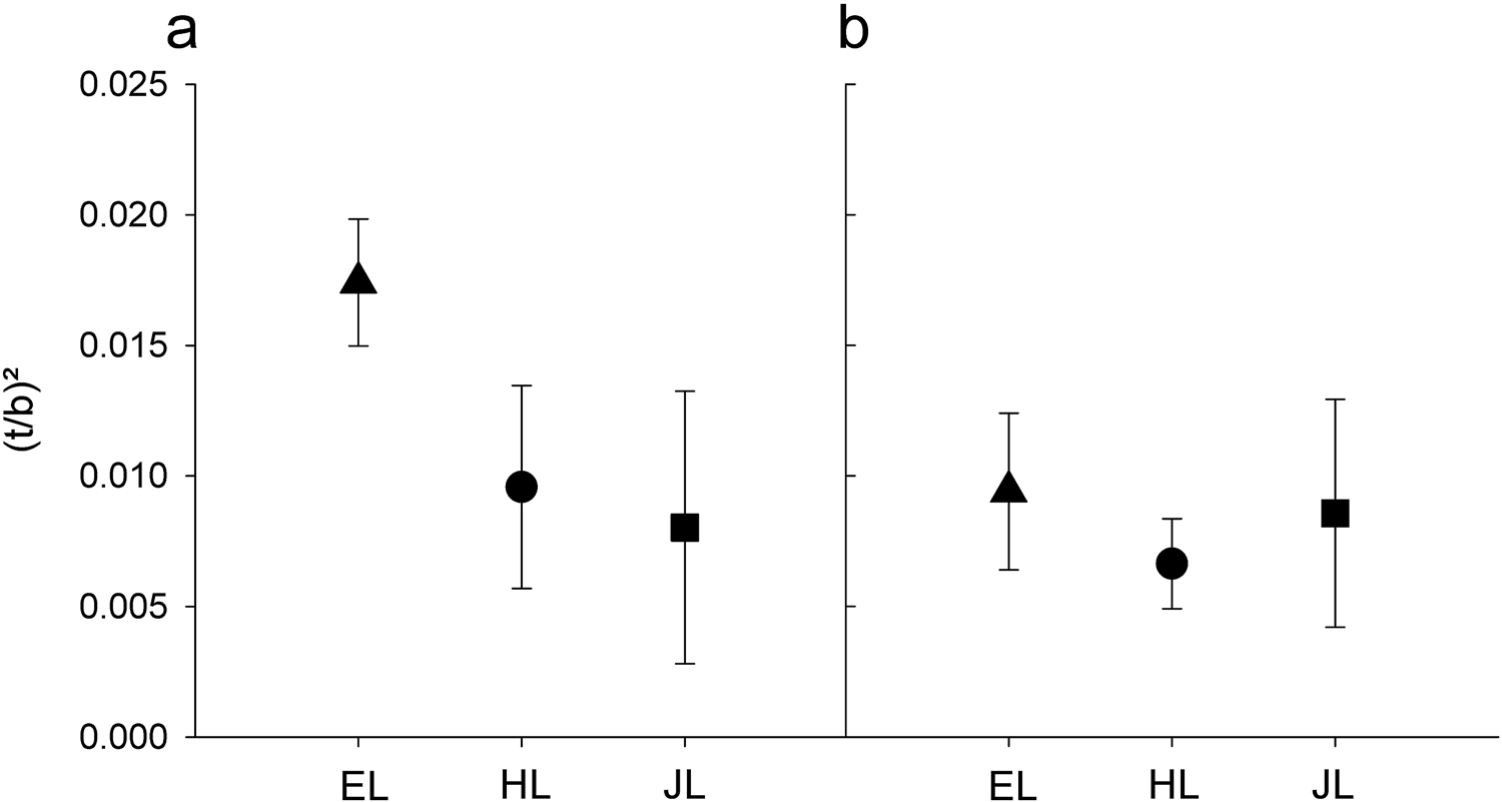
Conduit wall reinforcement in the tangential direction (**a**) and in the radial direction (**b**) in the first formed earlywood tracheids of European larch (EL), hybrid larch (HL) and Japanese larch (JL). Whiskers indicate the standard deviation (*n * = 6 trees/species)

In the wood formed before the start of the experimentation, we found no significant differences between Japanese larch (JL) and hybrid larch (HL) in lumen diameter or cell wall thickness traits (Table 4). However, European larch (EL) had significantly smaller tangential lumen diameters, which implied higher theoretical hydraulic safety against implosion of the tangential cell walls ((*t*_t_/*b*_t_)^2^) compared to the other two species (Fig. 6a, Table 4). The conduit wall reinforcement of the radial cell walls ((*t*_r_/*b*_r_)^2^) (Fig. 6b) showed no significant differences between species. The mean tangential lumen diameter was positively related to the initial biomass (*r* = 0.80, *P* < 0.0001, *n* = 18) across species (Online Resource 4a). Accordingly, (*t*_t_/*b*_t_)^2^ was negatively related to the initial biomass (*r* = 0.60, *P* < 0.01, *n* = 18) (Online Resource 4b).

**Table 4:** Means and standard errors of anatomical traits for three larch species; letters represent significant differences in the mean values at the p < 0.05 level for either species (n = 6 trees/species) or treatment (n =  9 trees/treatment)

Trait	Unit	Species	European larch	Hybrid larch	Japanese larch	Control	Drought stressed	Int
		*P*						*P*
*b*_r_	µm	*	25.37 ± 0.92b	29.41 ± 0.57a	26.52 ± 1.02ab	26.50 ± 0.63a	27.70 ± 1.08a	–
*b*_t_	µm	***	19.18 ± 0.77b	22.06 ± 0.31a	23.81 ± 0.42a	21.68 ± 0.78a	21.69 ± 0.81a	–
*t*_r_	µm	–	2.30 ± 0.13a	2.28 ± 0.11a	2.19 ± 0.17a	2.31 ± 0.10a	2.21 ± 0.12a	–
*t*_t_	µm	–	2.39 ± 0.12a	2.03 ± 0.15a	1.93 ± 0.22a	2.21 ± 0.14a	2.02 ± 0.16a	–
(*t*_r_/*b*_r_)^2^			0.0094 ± 0.0012a	0.0066 ± 0.0007a	0.0086 ± 0.0019a	0.0088 ± 0.0011a	0.0076 ± 0.0011a	*
(*t*_t_/*b*_t_)^2^		**	0.0174 ± 0.0010a	0.0096 ± 0.0016b	0.0080 ± 0.0021b	0.0125 ± 0.0019a	0.0108 ± 0.0019a	–
PMD	µm	–	14.71 ± 0.71a	15.27 ± 0.33a	15.80 ± 0.30a	15.28 ± 0.48a	15.24 ± 0.33a	–
TD	µm	–	7.94 ± 0.46a	8.31 ± 0.12a	8.71 ± 0.19a	8.27 ± 0.31a	8.37 ± 0.21a	–
AD	µm	–	4.53 ± 0.18a	4.85 ± 0.17a	4.57 ± 0.21a	4.78 ± 0.16a	4.53 ± 0.14a	–
TO		–	0.420 ± 0.036a	0.416 ± 0.022a	0.475 ± 0.021a	0.417 ± 0.026a	0.457 ± 0.019a	–
PC	µm^2^	**	43.50 ± 1.30b	48.24 ± 0.69a	47.59 ± 0.91a	46.08 ± 1.06a	46.81 ± 1.09a	–

Significant differences in the mean values at the *P* < 0.05 level for either species or treatment are indicated by different letters

Abbreviations are explained in Table 1

Species and species x treatment effects (Int) are indicated with “-” for *P* ≥ 0.5, “*” for *P* < 0.05, ** for *P* ≤ 0.01 and “***” for *P* ≤ 0.001. We found neither an effect of initial biomass nor of the treatment on the constitutive anatomical traits

There were no significant differences in pit membrane-, aperture-, and torus diameters as well as in the torus overlap between the species. Pit cavity area was, however, significantly smaller in EL than in HL or JL (Table 4).

## Discussion

### Influence of initial biomass on species specific transpiration and water use efficiency

The known large differences in growth rate between European larch (EL), Japanese larch (JL) and hybrid larch (HL) (Pâques et al. 2013) render experimentation as well as interpretation of biomass data and constitutive wood anatomy (Anfodillo et al. 2013; Piermattei et al. 2020) of young saplings challenging. Using younger HL plants resulted in an intermediary starting biomass, instead of much larger individuals than the parental species if older HL would have been used. The variation within the initial biomass, and thus the size of the plants, clearly had a significant effect on the final total needle surface, on the transpiration rate and on the relative biomass increase. Including the initial biomass into the ANCOVA allowed taking into account the size differences among plants. Still, relative biomass increase was significantly higher for HL than for EL, even though HL saplings were only in their 3rd growing season, compared to EL, which was in its 4th growing season. The faster growth of HL compared to one or both of its parents is generally observed (Pâques et al. 2013; Greenwood et al. 2015) but depends on parental varieties used. In sites, where (summer) water supply is not limited, JL growth can be close to that of HL (Philippe et al. 2016).

Transpiration rate per leaf surface was highest for EL and lowest for JL. The latter result is similar to a tendency for higher stomatal conductance, shown by Matyssek and Schulze (1987) for EL compared to JL, with HL showing intermediate values. Initial biomass clearly affected needle surface area and eventually total plant transpiration rate but we found no statistically significant impact on transpiration efficiency, suggesting that the differences in initial biomass did not affect the relative amounts of biomass growth vs. transpiration. As far as we know, there is no comparison of transpiration efficiency (TE) estimates either for EL and JL or their hybrid in the literature. The only estimate for a *Larix* species was for *L. occidentalis* with a TE at 3.6 g/kg (Marshall and Zhang 1994). For *Pseudotsuga menziesii*, which is in the same subfamily Laricoidae as *Larix*, Smit and van den Driessche (1992) estimated a TE of 6.2 g/kg. For other species from the Pinaceae family, low TE around 2.5 g/kg have been estimated for different *Abies* species (Becker 1977), whereas Guehl et al. (1995) estimated a TE of 5.3 g/kg to 6.6 g/kg for *Pinus pinaster*. Compared to the latter estimates, EL saplings (4.0 g/kg) appeared to have a medium, and JL saplings (6.1 g/kg) a rather high TE. Accordingly, leaf matter *δ*^13^C values were more negative for EL (suggesting lower WUE at leaf level), medium for HL and less negative for JL. This again is similar to the results from Matyssek and Schulze (1987), who showed a tendency for higher WUE of HL and JL compared to EL at the leaf level. Kloeppel et al. (1998) compared five different *Larix* species, including *L. decidua*, to a second sympatric evergreen conifer species growing on the same site, and all *Larix* species showed a tendency towards more negative *δ*^13^C values, thereby suggesting a lower WUE at the leaf level. Similarly, Gower and Richards (1990) showed more negative *δ*^13^C values for *L. occidentalis* and *L. lyallii* also compared to sympatric evergreen conifers (*Pinus*, *Abies* or *Tsuga* species). Overall, our results corroborate that EL has a relatively low WUE compared to HL and JL (but also compared to other conifer species) due to a higher transpiration or stomatal conductance per leaf surface and also lower growth.

### Species specific physiological responses to drought

There are no detailed ecophysiological studies comparing drought responses of the two parental *Larix* species and their hybrid; however, the drought response of EL has been regularly compared to other conifer species (e.g., Eilmann and Rigling 2012; Schuster and Oberhuber 2013; Peters et al 2019). Eilmann and Rigling (2012) showed that the growth of EL was fairly low during drought, and that EL lacked the ability to recover from drought. This was confirmed by Schuster and Oberhuber (2013), who showed a stronger decline in the basal area index of EL (compared to *P. abies* and *P. sylvestris*) in response to a decrease in soil moisture. Similarly, Lévesque et al. (2013, 2014a) showed that EL was more vulnerable to drought (in terms of ring growth) than *P. sylvestris* and *Pinus nigra*. Overall, the literature suggests that the growth of EL is strongly affected by soil drought (George et al. 2016). The drought stress applied in our study had a significant effect on stem diameter (radial growth) in all species, but a lower absolute biomass increment and relative biomass was only significant in JL and even more so in HL. These results are in line with a stronger dependence of HL ring growth to precipitation compared to EL, as observed by Oleksyn and Fritts (1991). Marchal et al. (2019) had shown a stronger plasticity of HL in response to soil water deficit in terms of ring width compared to EL and JL, where more stressful conditions did decrease its level of performances to a level comparable to parental species. In our study, only EL showed no significant reduction in transpiration rate under drought. In contrast, HL and JL had a significant decrease in transpiration, which was proportionally higher compared to biomass increase, resulting in an increase in transpiration efficiency with drought. Lévesque et al. (2014b) showed highest increase in WUE between a mesic and a xeric site for EL, compared to evergreen conifer species. Here, the strong drought effect on growth for HL was mainly due to a strong reduction in the transpiration rate, suggesting a higher stomatal sensitivity of HL to drought. Drought stressed saplings of JL showed a significant decrease in needle surface area, whereas HL did show a quite similar needle surface area in control and stressed trees. Maintenance of a large leaf surface by HL under drought can explain the observed severe reduction in transpiration rate and, therefore, implies stronger stomatal control of HL. Thus, a significant increase of transpiration efficiency was only observed for HL and JL, the increase being much stronger for JL. These results suggest that EL was better able to maintain stomatal opening under soil water deficit and thus maintain photosynthesis. At the leaf level, HL showed the biggest shift in *δ*^13^C values (nearly 2‰) under drought, indicating either strong stomatal closure or an increase in photosynthesis. As the latter is unlikely under drought conditions, the *δ*^13^C measurements confirm the strong stomatal reaction to drought by HL. Several authors suggest an anisohydric stomatal behaviour of EL in response to soil water deficit; compared to evergreen conifer species (Anfodillo et al. 1998; Swidrak et al. 2013; Klein 2014; Leo et al. 2014), it is able to maintain high transpiration under drought conditions, thus keeping stomata open (Streit et al. 2014) even at very low levels of leaf water potential. We confirm this behaviour at the whole plant level, as EL maintained its transpiration rate under drought. Furthermore, the strong reduction in transpiration of HL under drought would indicate its isohydric behaviour to increasing soil water deficit. However, during a mild drought stress, anisohydric behaviour of HL might be possible, since HL solely showed a significant decrease in relative water content in sapwood under the impact of prolonged drought stress. The observed relative water loss of 15% in drought stressed HL corresponds to more than 40% of loss in hydraulic conductivity (Rosner et al. 2019), which is quite considerable for conifers. Bhusal et al. 2020 recently reported that JL shows both isohydric and anisohydric drought response, however, with a stronger tendency towards isohydry.

In response to drought, not only the production of the wood volume is reduced, but anatomical traits, such as the diameter and cell wall thickness of tracheids, are also affected. Reduction in lumen diameters and an increase in cell wall thickness are important anatomical features for a trees’ drought adaptability, because higher wall/lumen relationships would promote greater mechanical support to stems while preventing xylem cell collapse (Hacke et al. 2001; Domec et al. 2008; Rosner et al. 2018). Towards the end of the drought stress experiment, lumen diameters in the sapwood of stressed HL and JL decreased much more than in EL. In HL, a trend in cell wall thickness increase was also observed. These structural modifications indicate either an earlier production of transition wood, which has a higher hydraulic safety than earlywood and latewood (Dalla-Salda et al. 2014) or intra-annual density fluctuations such as the formation of a “false ring”. Since the decrease in radial lumen diameter was more observed in individuals with higher *δ*^13^C and transpiration efficiency, we suggest that anatomical modifications were triggered by physiological processes such as the earlier closure of the stomata. Intra-annual density variations in earlywood (“false rings”) develop under the impact of early summer drought. When water is again available, the tree starts to produce wood that resembles earlywood (Fritts 1976; Wimmer et al. 2000; Rosner et al. 2018; George et al. 2019). Sugar investment for cell wall thickening and lignification exceeds other growth processes (Cartenì et al. 2018) and the production of “false rings” that resemble latewood cells in their cell wall thickness might be related to biomechanical demands of the tree. Concerning the production of “false rings”, HL seems to be the most sensitive of the investigated larch species and if this behaviour is relevant for recovery after drought, further investigation in this context is needed.

### Investment in hydraulic safety impacts reaction to drought in larch species

Structural modifications in wood developed to prevent or minimize cavitation include: pit structure (Pittermann et al. 2006; Delzon et al. 2010; Bouche et al. 2014), conduit wall reinforcement by decreasing lumen diameter or by increasing wall thickness (Hacke et al. 2001; Domec et al. 2009) and possibly also differences in cell wall lignification (Rosner et al. 2018). One of the anatomical traits that shows a positive correlation with embolism resistance is the conduit wall reinforcement, i.e., the cell wall thickness to span ratio (*t*/*b*)^2^, because it influences the resistance against hydraulic failure (Hacke et al. 2001). For example, in mature *Larix decidua* trunks, (*t*/*b*)^2^ values of earlywood increase toward the apex, where water potential is known to become more negative (Prendin et al. 2017). In our study, there were differences among species in (*t*/*b*)^2^ measured in the tangential direction of non-stressed sapwood, with EL showing significantly higher values than HL and JL. This suggests that the hydraulic safety of EL was superior to the other two species in terms of resistance against cavitation (Rosner et al. 2016), which would allow for the observed higher transpiration per leaf area under drought for EL. Higher growth, as generally observed in juvenile HL and JL (Pâques et al. 2013; Caudullo et al. 2018), comes at the cost of lower hydraulic safety, because tracheids become bigger (and thus more prone to cavitation) with distance from the apex (Anfodillo et al. 2013; Piermattei et al. 2020). To take into account such structure–function relationships, future experiments should include same-age as well as same-size saplings across species, so that age and size effects can be clearly separated from species differences.

The significantly smaller pit cavities we observed for EL compared to HL and JL might be related to higher hydraulic safety; however, this anatomical trait has so far not been tested as a proxy for vulnerability to cavitation. Pit anatomy plays an important role in cavitation resistance and the most commonly used proxy is the torus overlap (Delzon et al. 2010; Bouche et al. 2014). Torus overlap values obtained in our study suggest that juvenile larch wood is hydraulically quite safe when compared to other conifer species (Bouche et al. 2014). We found a trend for higher torus overlap in JL followed by EL and HL. However, regarding conduit wall reinforcement in the tangential direction (Rosner et al. 2016), JL was the most cavitation sensitive species. The higher torus overlap might be thus a compromise between hydraulic efficiency and hydraulic safety in this species.

Overall, EL had higher hydraulic safety, in terms of conduit wall reinforcement and pit cavity size, than JL and HL, which comes at the cost of slower growth. This corresponds to the classification of EL as an anisohydric species (Anfodillo et al. 1998; Swidrak et al. 2013; Klein 2014; Leo et al. 2014), which can cope with lower water potentials than isohydric species (McDowell et al. 2008), but their wood must be constructed more safely to avoid implosion (Hacke et al. 2001). From the constitutive structure–function point of view, wood of EL is designed to keep stomata open at more negative water potentials than JL or HL, because cavitation occurs at lower water potentials. Accordingly, EL had a lower conductivity loss, indicated by a tendency to higher RWC (Rosner et al. 2019), compared to JL and HL.

## Conclusions

Our study showed that young saplings of the three larch species (EL, JL and HL) adopted different strategies towards drought. As hypothesised, the slower growth and hydraulically safer wood of EL allows anisohydric behaviour under drought stress, whereas the less safe wood design in JL and HL demands stronger stomatal control and an isohydric strategy. Both JL and HL had an increase in transpiration efficiency induced by drought, but in HL, the increase was due to a strong reduction in transpiration per leaf surface, whereas in JL it was due to a reduction in the leaf surface. HL was the most reactive to soil water availability; it showed the highest decrease in transpiration rate, had significantly lower relative water contents in sapwood and started to produce denser wood much earlier than the other two species. We hypothesised higher drought plasticity during growth in HL, for now, we cannot reject this hypothesis, but we conclude for the HL analysed in the present study, that their strategy towards drought stress was inherited from JL rather than from EL. The anisohydric drought response strategy of EL could allow a range shift under climate change from its native range to higher elevations or more northern regions. The observed higher growth of HL, even under drought, and its higher resistance against diseases, supports its inclusion in pure and mixed lowland plantations in regions, where EL is native. HL is a “man-made” construction obtained by controlled crosses between both parents selected for their superiority. Using connected pedigrees at intra- and inter-specific levels through crossing of common parents such as in diallel mating design would allow drawing a clearer conclusion on the genetic determinism of the observed differences between hybrids and parental species. To investigate a species’ strategy for its response to drought we suggest both quantitative anatomical and physiological investigations, whereby the relative water loss of the sapwood is a fast and easily assessable functional trait.

### Author contribution statement

LEP provided plant material; OB set up the green house experiment and supervised GB, who performed the physiological experiments. GB wrote the first report on the physiology results. SR, AS and NG were responsible for the anatomical trait dataset design and supervision of NS, who did the practical histological work. OB performed statistical data analyses for all traits. SR and NS did statistical data analyses for selected anatomical traits. NS and SR produced the figure plots. The first draft of the manuscript was written by NS and AS. SR, OB, LEP and NG reviewed this draft in several rounds. After input of the editor and two reviewers, all authors contributed to two revised versions. All authors agree on the content of this manuscript.

## Supplementary Information

Below is the link to the electronic supplementary material.

Supplementary file1 (PPTX 1698 kb)

## Data Availability

Datasets will be provided to colleagues on demand.

## References

[CR1] Allen CD, Breshears DD, McDowell NG (2015). On underestimation of global vulnerability to tree mortality and forest die-off from hotter drought in the Anthropocene. Ecosphere.

[CR2] Anfodillo T, Rento S, Carraro V, Furlanetto L, Urbinati C, Carrer M (1998). Tree water relations and climatic variations at the alpine timberline: seasonal changes of sap flux and xylem water potential in *Larix decidua* Miller, *Picea abies* (L.) Karst, and *Pinus cembra* L. Ann Des Sci For.

[CR3] Anfodillo T, Petit G, Crivellaro A (2013). Axial conduit widening in woody species: a still neglected anatomical pattern. IAWA J.

[CR4] Becker M (1977). Contribution to the study of transpiration and adaptation to drought in young conifers: Studies on three circum-mediterranean firs (*Abies alba*, *A. nordmanniana* and *A. numidica*)”. Ann Sci Forest.

[CR5] Beikircher B, Ameglio T, Cochard H, Mayr S (2010). Limitation of the Cavitron technique by conifer pit aspiration. J Exp Bot.

[CR6] Bhusal N, Lee M, Reum Han AR, Han A, Kim HS (2020). Responses to drought stress in *Prunus sargentii* and *Larix kaempferi* seedlings using morphological and physiological parameters. Forest Ecol Manag.

[CR7] Bogeat-Triboulot MB, Buré C, Gerardin T, Chuste PA, Le Thiec D, Hummel I, Durand M, Wildhagen H, Douthe C, Molins A, Galmés J, Smith HK, Flexas J, Polle A, Taylor G, Brendel O (2019). Additive effects of high growth rate and low transpiration rate drive differences in whole plant transpiration efficiency among black poplar genotypes. Environ Exp Bot.

[CR8] Bouche PS, Larter M, Domec J-C, Burlett R, Gasson P, Jansen S, Delzon,  (2014). A broad survey of hydraulic and mechanical safety in the xylem of conifers. J Exp Bot.

[CR9] Boudru M. 1986. Forêt et silviculture. Sylviculture appliquée. Ed. Presses Agronomiques de Gembloux. Gembloux. 122–131.

[CR10] Breda N, Huc R, Granier A, Dreyer E (2006). Temperate forest trees and stands under severe drought: a review of ecophysiological responses, adaptation processes and long-term consequences. Ann For Sci.

[CR11] Cartenì F, Deslauriers A, Rossi S, Morin H, De Micco V, Mazzoleni S, Giannino F (2018). The physiological mechanisms behind the earlywood-to-latewood transition: a process-based modeling approach. Front Plant Sci.

[CR12] Caudullo G., Nakada R., Da Ronch F. 2018. *Larix kaempferi* (Lambert) Carrière, 1856. III. Monographien von Baum‐ und Straucharten. 1. Nadelbaumarten der temperierten Klimazonen. Enzyklopädie der Holzgewächse – 70. Erg. Lfg. 01/18. 10.1002/9783527678518.ehg2017003

[CR13] Cazaux JP, Chevalier R, Gilbert JM, Ginisty C (1993) Le mélèze hybride en plantation: résultats provisoires sur 17 sites en France. Informations techniques du Cemagref nr 91:8. https://hal.inrae.fr/hal-02576271. Assessed on 26 Mar 2021

[CR14] Dalla-Salda G, Fernández ME, Sergent A-S, Rozenberg P, Badel E, Martinez-Meier A (2014). Dynamics of cavitation in a Douglas-fir tree-ring: transition-wood, the lord of the ring?. J Plant Hydraul.

[CR15] Delzon S, Douthe C, Sala A, Cochard H (2010). Mechanism of water-stress induced cavitation on conifers: bordered pit structure and function support the hypothesis of seal capillary-seeding. Plant Cell Environ.

[CR16] Dietrich L, Delzon S, Hoch G, Kahmen A (2019). No role for xylem embolism or carbohydrate shortage in temperate trees during the severe 2015 drought. J Ecol.

[CR17] Domec JC, Lachenbruch B, Meinzer FC, Woodruff DF, Warren JM, McCulloh KA (2008). Maximum height in a conifer is associated with conflicting requirements for xylem design. Proc Natl Acad Sci (USA).

[CR18] Domec JC, Warren JM, Meinzer FC, Lachenbruch B (2009). Safety factors for xylem failure by implosion and air-seeding within roots, trunks and branches of young and old conifer trees. IAWA J.

[CR19] Eilmann B, Rigling A (2012). Tree-growth analyses to estimate tree species drought tolerance. Tree Physiol.

[CR20] Feichtinger LM, Eilmann B, Buchmann N, Rigling A (2014). Growth adjustments of conifers to drought and to century-long irrigation. For Ecol Managem.

[CR21] Fritts H (1976). Tree rings and climate.

[CR22] Geburek T (2002) *Larix decidua*. III. Monographien von Baum‐ und Straucharten. 1. Nadelbaumarten der temperierten Klimazonen. Enzyklopädie der Holzgewächse – 29. Erg. Lfg. 9/02

[CR23] George J-P, Grabner M, Karanitsch-Ackerl S, Mayer K, Weißenbacher L, Schueler S (2016). Genetic variation, phenotypic stability, and repeatability of drought response in European larch throughout 50 years in a common garden experiment. Tree Physiol.

[CR24] George J-P, Grabner M, Campelo F, Karanitsch-Ackerl S, Mayer K, Klumpp RT, Schueler S (2019). Intra-specific variation in growth and wood density traits under water-limited conditions: Long-term-, short-term-, and sudden responses of four conifer tree species. Sci Total Environ.

[CR25] Gower ST, Richards JH (1990). Larches: deciduous conifers in an evergreen world. Bioscience.

[CR26] Granier A, Bréda N, Biron P, Villette S (1999). A lumped water balance model to evaluate duration and intensity of drought constraints in forest stands’. Ecol Modeling.

[CR27] Greenwood MS, Roth BE, Maass D, Irland LC (2015). Near rotation-length performance of selected hybrid larch in Central Maine, USA. Silvae Genet.

[CR28] Guehl J-M, Nguyen-Queyrens A, Loustau D, Ferhi A (1995) Genetic and environmental determinants of water-use efficiency and carbon isotope discrimination in forest trees. In: Sandermann H, Bonnet-Masimbert M (eds) Eurosilva: Contribution to forest tree physiology. results from eurosilva projects. les colloques. Editions Colloques de l’INRA, Paris, pp 297–321. https://hal.inrae.fr/hal-02779528. Assessed on 26 Mar 2021

[CR29] Haasemann W (1986). Untersuchungen zur Ökologie der Europäerlärche, Japanerlärche und ihrer Hybriden im Nass-Trockenfeld. Beitr Forstwirschaft.

[CR30] Hacke UG, Sperry JS, Pockman WT, Davis SD, McCulloch KA (2001). Trends in wood density and structure are linked to prevention of xylem implosion by negative pressure. Oecologia.

[CR31] Huang WW, Fonti P, Larsen JB, Raebild A, Callesen I, Pedersen NB, Hansen JK (2017). Projecting tree-growth responses into future climate: A study case from a Danish-wide common garden. Agric For Meteor.

[CR32] Jansen S, Geburek T (2016). Historic translocations of European larch (*Larix decidua* Mill.) genetic resources across Europe—a review from the 17th until the mid-20th century. Forest Ecol Managem.

[CR33] Klein T (2014). The variability of stomatal sensitivity to leaf water potential across tree species indicates a continuum between isohydric and anisohydric behaviours. Funct Ecol.

[CR34] Klein T, Cahanovitc R, Sprintsin M, Herr N, Schiller G (2019). A nation-wide analysis of tree mortality under climate change: Forest loss and its causes in Israel 1948–2017. For Ecol Manag.

[CR35] Kloeppel BD, Gower ST, Treichel IW, Kharuk S (1998). Foliar carbon isotope discrimination in *Larix* species and sympatric evergreen conifers: A global comparison. Oecologia.

[CR36] Leo M, Oberhuber W, Schuster R, Grams TEE, Matyssek R, Wieser G (2014). Evaluating the effect of plant water availability on inner alpine coniferous trees based on sap flow measurements. Eur J For Res.

[CR37] Lévesque M, Saurer M, Siegwolf R, Eilmann B, Brang P, Bugmann H, Rigling A (2013). Drought response of five conifer species under contrasting water availability suggests high vulnerability of Norway spruce and European larch. Global Change Biol.

[CR38] Lévesque M, Rigling A, Bugmann H, Weber P, Brang P (2014). Growth response of five co-occurring conifers to drought across a wide climatic gradient in central Europe. Agric For Meteor.

[CR39] Lévesque M, Siegwolf R, Saurer M, Eilmann B, Rigling A (2014). Increased water-use efficiency does not lead to enhanced tree growth under xeric and mesic conditions. New Phytol.

[CR40] Marchal A, Schlichting CD, Gobin R, Balandier P, Millier F, Munoz F, Pâques LE, Sanchez Rodriguez L (2019). Deciphering hybrid larch reaction norms using random regression. G3 Genes Genomes Genet.

[CR41] Marshall JD, Zhang JW (1994). Carbon-isotope discrimination and water-use efficiency in native plants of the north central Rockies. Ecology.

[CR42] Masson G (2005). Autoécologie des essences forestières.

[CR43] Matyssek R, Schulze E-D (1987). Heterosis in hybrid larch (*Larix decidua* x *leptolepis*). II Growth characteristics. Trees.

[CR44] McDowell NG, Allen CD (2015). Darcy's law predicts widespread forest mortality under climate warming. Nat Clim Change.

[CR45] McDowell N, Pockman WT, Allen CD, Breshears DD, Cobb N, Kolb T, Plaut J, Sperry J, West A, Williams DG, Yepez EA (2008). Mechanisms of plant survival and mortality during drought: why do some plants survive while others succumb to drought?. New Phytol.

[CR46] McDowell NG, Williams AP, Xu C, Pockman WT, Dickman LT, Sevanto S, Pangle R, Limousin J, Plaut J, Mackay DS, Ogée J, Domec J-C, Allen CD, Fisher RA, Jiang X, Muss JD, Breshears DD, Rauscher SA, Koven C (2016). Multi-scale predictions of massive conifer mortality due to chronic temperature rise. Nat Clim Change.

[CR47] Mencuccini M, Binks O (2015). Tall leafy conifers lose out. Nat Clim Change.

[CR48] Nagamitsu T, Matsuzaki T, Nagasaka K (2018). Provenance variations in stem productivity of 30-year-old Japanese larch trees planted in northern and central Japan are associated with climatic conditions in the provenances. J For Res.

[CR49] Obojes N, Meurer A, Newesely C, Tasser E, Oberhuber W, Mayr S, Tappeiner U (2018). Water stress limits transpiration and growth of European larch up to the lower subalpine belt in an inner-alpine dry valley. New Phytol.

[CR50] Oleksyn J, Fritts HC (1991). Influence of climatic factors upon tree rings of *Larix decidua* and *L. decidua* × *L. kaempferi* from Pulawy. Poland Trees.

[CR51] Pâques LE, Foffová E, Heinze B, Lelu-Walter MA, Liesebach M, Philippe G (2013) Larches (*Larix* sp.). In: Pâques, L.E. (eds.) Forest tree breeding in Europe. Managing Forest Ecosystems, 25. Springer, Dordrecht, p 52

[CR52] Peters RL, Speich M, Pappas C, Kahmen A, von Arx G, Graf Pannatier E, Steppe K, Treydte K, Stritih A, Fonti P (2019). Contrasting stomatal sensitivity to temperature and soil drought in mature alpine conifers. Plant Cell Environ.

[CR53] Philippe G, Buret C, Matz S, Pâques LE (2016). Composition of hybrid larch (*Larix* × *eurolepis* Henry) forest reproductive materials: How much does hybrid percentage affect stand performance?. New For.

[CR54] Piermattei A, von Arx G, Avanzi C, Fonti P, Gärtner H, Piotti A, Urbinati C, Vendramin GG, Büntgen U, Crivellaro A (2020). Functional relationships of wood anatomical traits in Norway spruce. Front Plant Sci.

[CR55] Pittermann J, Sperry JS, Hacke UG, Wheeler JK, Sikkema EH (2006). Inter-tracheid pitting and the hydraulic efficiency of conifer wood: The role of tracheid allometry and cavitation protection. Am J Bot.

[CR56] Prendin AL, Petit G, Fonti P, Rixen C, Dawes MA, von Arx G (2017). Axial xylem architecture of *Larix decidua* exposed to CO2 enrichment and soil warming at the tree line. Funct Ecol.

[CR57] R Core Team (2020) A Language and environment for Statistical Computing. R Foundation for Statistical Computing, Vienna, Austria. https://www.r-project.org. Assessed on 26 Mar 2021

[CR58] Rosner S, Svetlik J, Andreassen K, Borja I, Dalsgaard L, Evans R, Luss S, Tveito OE, Solberg S (2016). Novel hydraulic vulnerability proxies for a boreal conifer species reveal that opportunists may have lower survival prospects under extreme climatic events. Front Plant Sci.

[CR59] Rosner S, Gierlinger N, Klepsch M, Karlsson B, Evans R, Lundqvist SO, Svetlik J, Borja I, Dalsgaard L, Andreassen K, Solberg S, Jansen S (2018). Hydraulic and mechanical dysfunction of Norway spruce sapwood due to extreme summer drought in Scandinavia. For Ecol Manag.

[CR60] Rosner S, Johnson DM, Voggeneder K, Domec J-C (2019). The conifer-curve: Fast prediction of hydraulic conductivity loss and vulnerability to cavitation. Ann For Sci.

[CR61] Rozenberg P, Chauvin T, Escobar-Sandoval M, Charpentier J-P, Pâques L (2020). Climate warming differently affects *Larix decidua* ring formation at each end of a French Alps elevational gradient. Ann For Sci.

[CR62] Schneck V, Schneck D, Grotehusmann H, Pâques LE (2002) Testing of hybrid larch over a broad range of site conditions. In: Improvement of larch (*Larix* sp.) for better growth, stem form and wood quality, Proceedings of the International Symposium Improvement of larch (*Larix* sp.) for better growth, stem form and wood quality, Gap, FRA, pp119–126. https://hal.inrae.fr/hal-02763854. Assessed on 26 Mar 2021

[CR63] Schuster R, Oberhuber W (2013). Drought sensitivity of three co-occurring conifers within a dry inner Alpine environment. Trees.

[CR64] Smit J, van den Driessche R (1992). Root Growth and Water Use Efficiency of Douglas-Fir (*Pseudotsuga menziesii* (Mirb.) Franco) and Lodgepole Pine (*Pinus Contorta* Dougl.) Seedlings. Tree Physiol.

[CR65] Streit K, Siegwolf RTW, Hagedorn F, Schaub M, Buchmann N (2014). Lack of photosynthetic or stomatal regulation after 9 years of elevated CO_2_ and 4 years of soil warming in two conifer species at the alpine treeline. Plant Cell Environ.

[CR66] Swidrak I, Schuster R, Oberhuber W (2013). Comparing growth phenology of co-occurring deciduous and evergreen conifers exposed to drought. Flora.

[CR67] Wimmer R, Strumia G, Holawe F (2000). Use of false rings in Austrian pine to reconstruct early growing season precipitation. Can J For Res.

