# Supplementary material for: Physiological and anatomical responses to drought stress differ between two larch species and their hybrid
Source: Trees (Berl West). 2021 May 7;35(5):1467–84. doi: 10.1007/s00468-021-02129-4 (PMC8550302; doi:10.1007/s00468-021-02129-4)
Supplement: Supplementary file 1 — Supplementary file1 (PPTX 1698 kb) [file 468_2021_2129_MOESM1_ESM.pptx]

## Slide 1
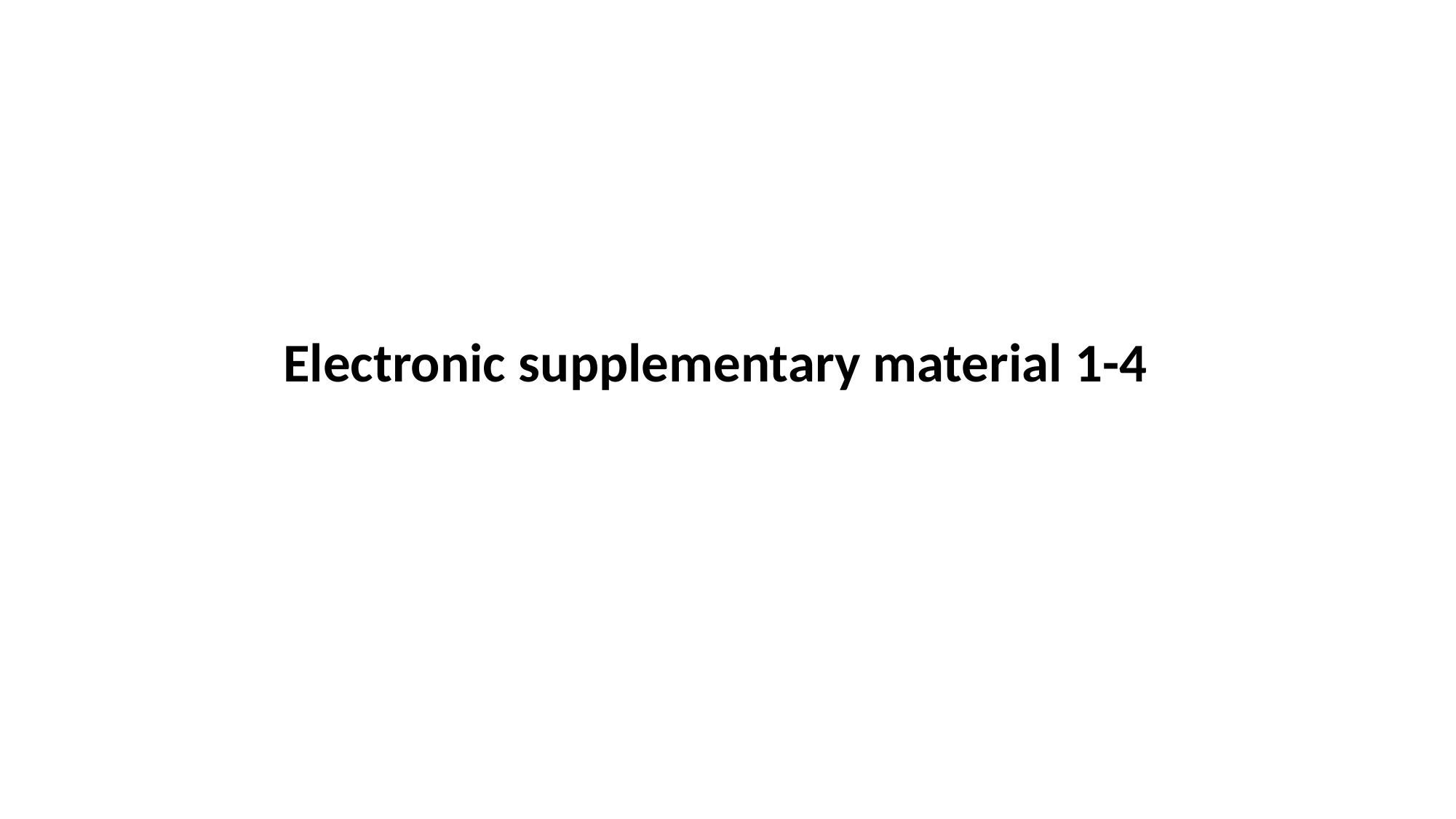

Electronic supplementary material 1-4

## Slide 2
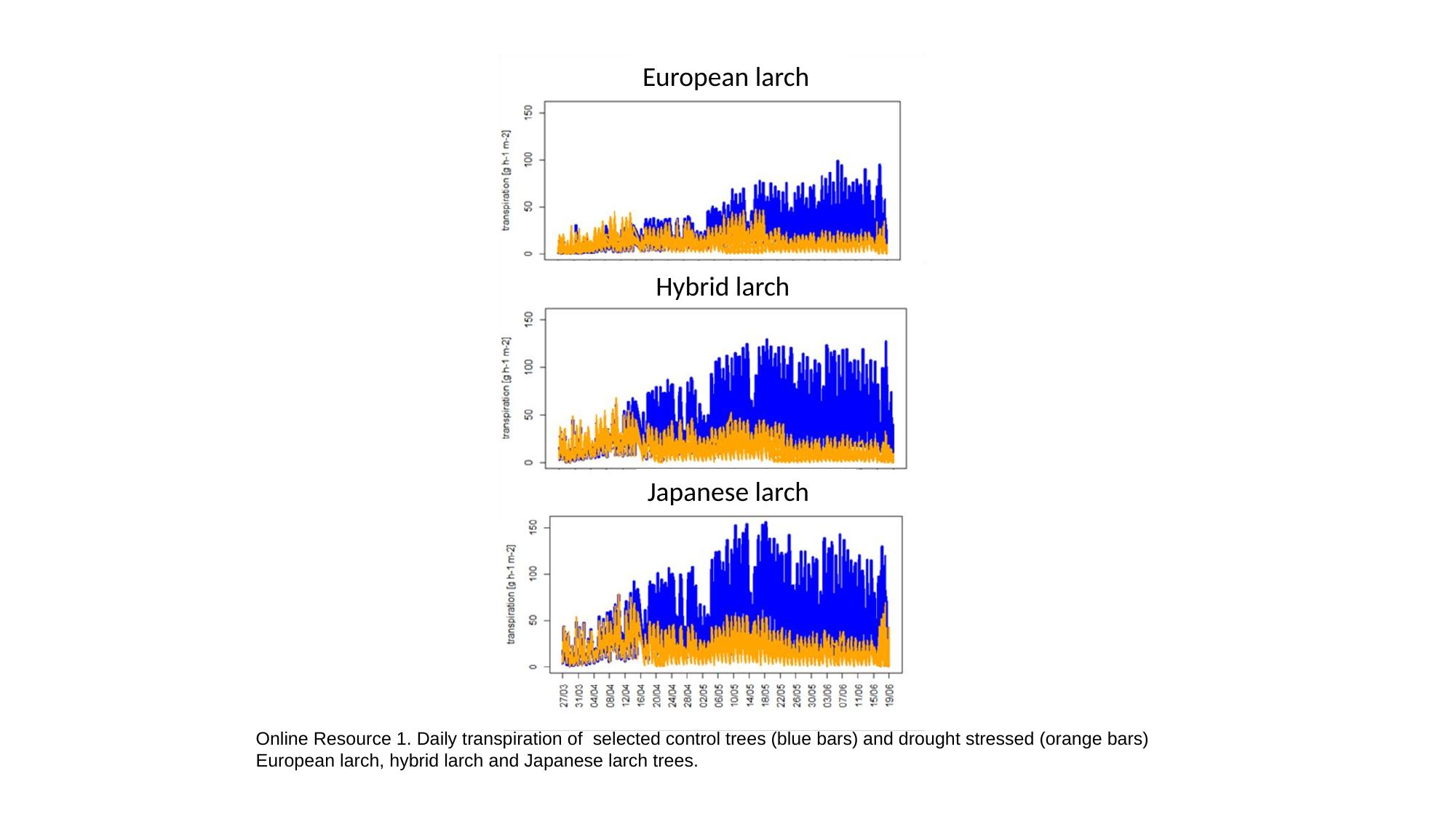

European larch
Hybrid larch
Japanese larch
Online Resource 1. Daily transpiration of selected control trees (blue bars) and drought stressed (orange bars) European larch, hybrid larch and Japanese larch trees.

## Slide 3
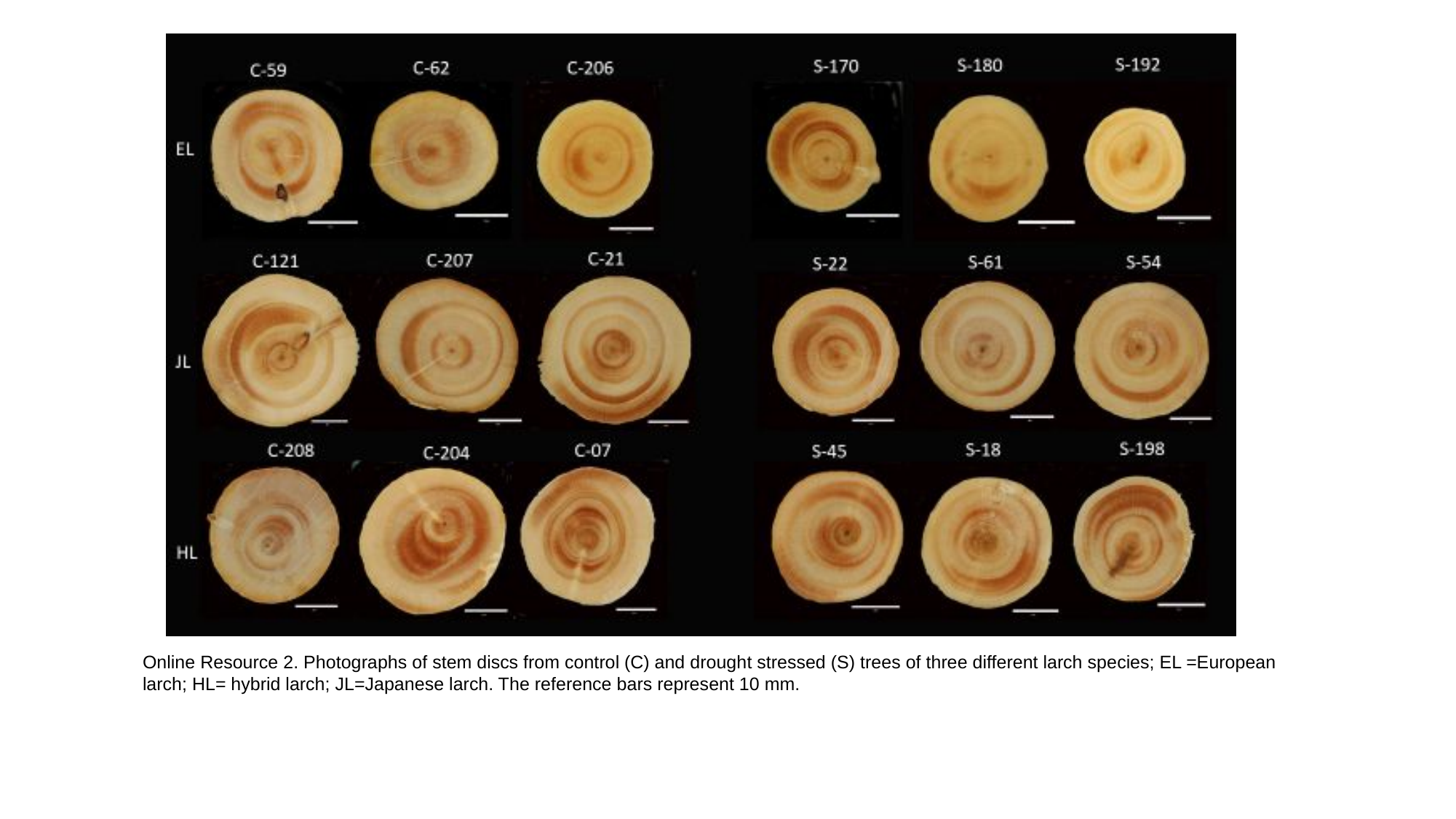

Online Resource 2. Photographs of stem discs from control (C) and drought stressed (S) trees of three different larch species; EL =European larch; HL= hybrid larch; JL=Japanese larch. The reference bars represent 10 mm.

## Slide 4
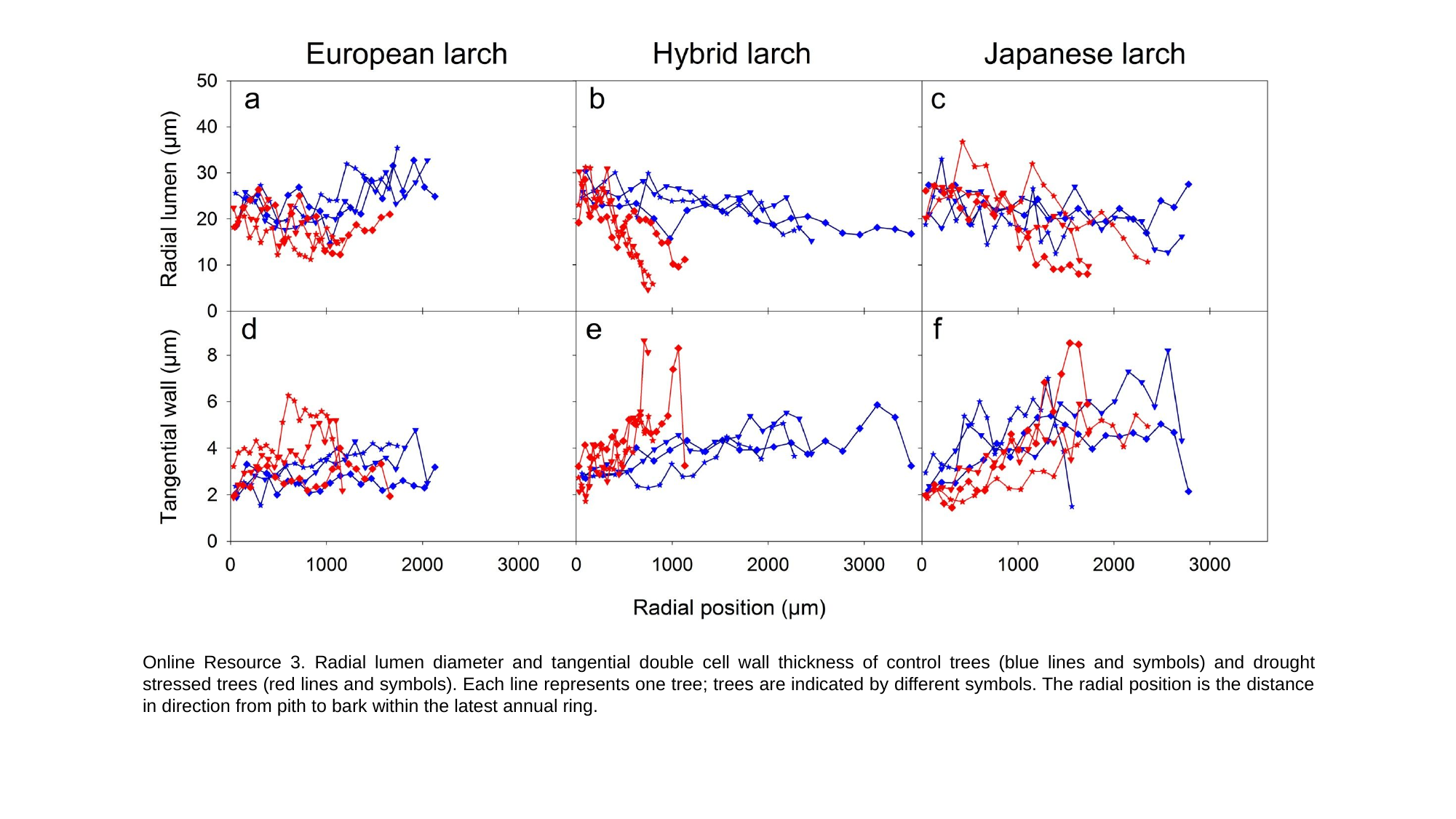

Online Resource 3. Radial lumen diameter and tangential double cell wall thickness of control trees (blue lines and symbols) and drought stressed trees (red lines and symbols). Each line represents one tree; trees are indicated by different symbols. The radial position is the distance in direction from pith to bark within the latest annual ring.

## Slide 5
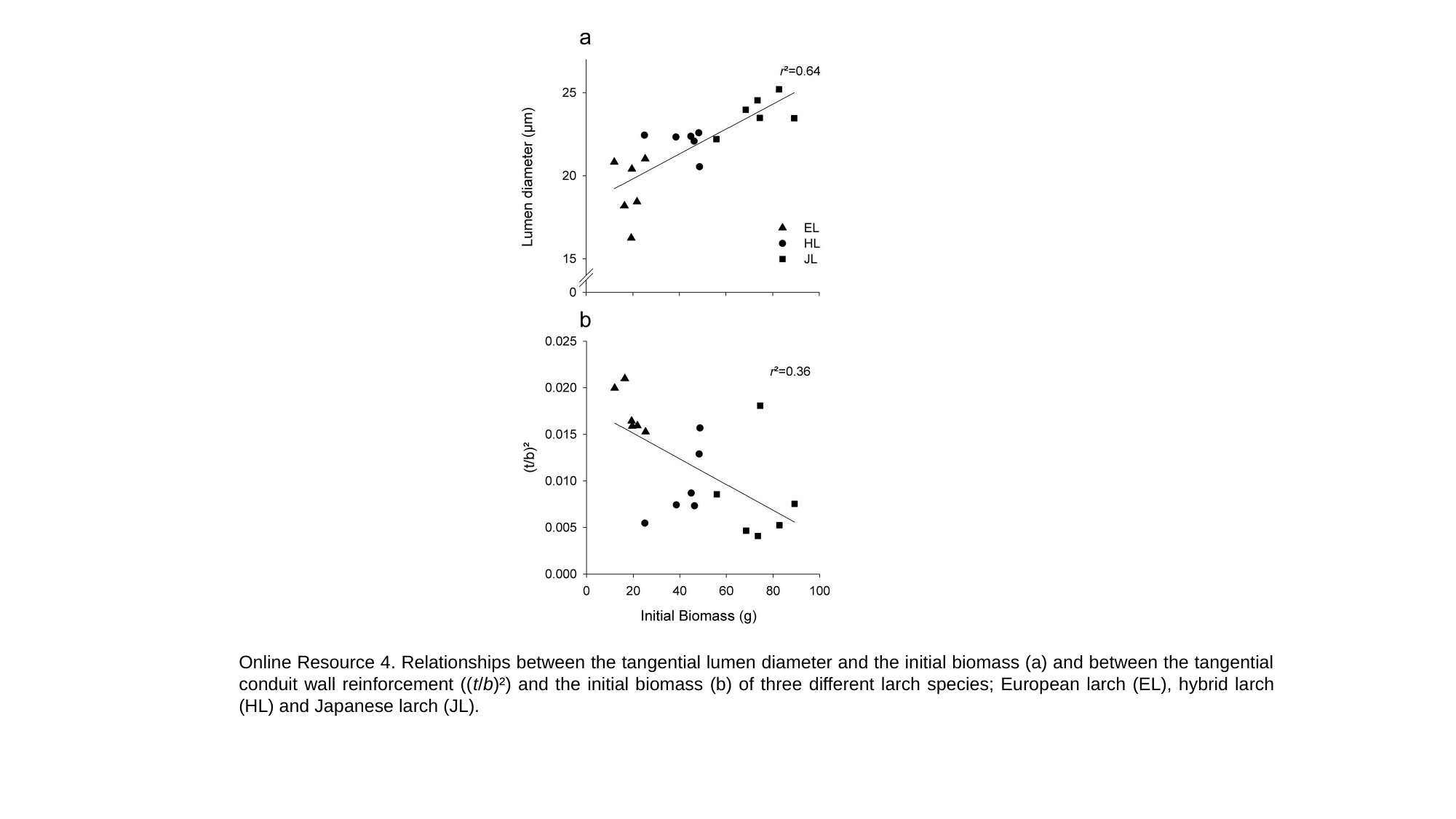

Online Resource 4. Relationships between the tangential lumen diameter and the initial biomass (a) and between the tangential conduit wall reinforcement ((t/b)²) and the initial biomass (b) of three different larch species; European larch (EL), hybrid larch (HL) and Japanese larch (JL).
